# 12th ISNS European Regional Meeting Oral and Poster Abstracts

**DOI:** 10.3390/ijns7040071

**Published:** 2021-10-29

**Authors:** Kate Hall

**Affiliations:** International Society for Neonatal Screening, Reigerskamp 273, 3607 HP Maarssen, The Netherlands; office-manager@isns-neoscreening.org

**Keywords:** newborn screening, ISNS, Europe

## Abstract

Due to the impact worldwide of COVID-19, the 12th European ISNS meeting planned to be live in Luxembourg in November 2020 became Luxembourg Going Virtual in November 2021. The conference theme derived from the geographic location of Luxembourg was retained: *Newborn screening—working together in the heart of Europe*. Abstracts of the newborn screening experience and knowledge shared in both oral presentations and posters at the symposium are gathered here to assist in selecting presenters to attend virtually and posters to view online. Some abstract highlights include findings from pilot studies of new screening disorders, the value of screening older previously unscreened children, and benefits of second tier testing.

## 1. Invited Presentations

I00. **Working Together in the Heart of Europe**

Jim R Bonham

President, International Society for Neonatal Screening

The last year and a half has been a remarkable time for countries across the world. We moved from an era of normal social contact into the virtual age in one giant leap and sadly almost a quarter of a billion people have contracted COVID-19 with around 5 million tragically dying as a result.

Of course babies have continued to be born and I must pay tribute to the way in which newborn screening programs across the world continued to operate and to the dedication of the staff—all of you, who have made this possible—and the industries, including our sponsors, who have maintained supplies in this difficult time.

During the last 18 months we learned much about mass genetic testing in our populations and this has emphasized the potential for the technology to be applied in public health programs and newborn screening may be a beneficiary of this. We shall hear much more of this during our conference. The months of isolation also reminded us to value one another and the human touch, to make our societies grow and flourish.

In an exciting development on 28 June 2021 saw the first ‘International Neonatal Screening Day’ which ISNS has helped create, and we look forward to this developing in 2022.

Within Europe itself, we have seen a growing emphasis, supported by on-line meetings, to work with policy makers, MEPs, patient groups, the European Reference Networks and of course ISNS to help develop screening policy and practice. You will be hearing more about this during the meeting.

Ultimately; however, it is science and medicine that delivers life changing benefits for our children and their families and looking at the program we have much to learn and much to celebrate during the coming days of this exciting three day conference, I hope that you enjoy the talks and unlock some of the potential that they contain.

I01. **Whole Genome Sequencing for Precision Diagnostics in Rare Diseases—Implications for Newborn Screening**

Anna Wedell

Karolinska Institute, Stockholm, Sweden

In 2015, whole genome sequencing (WGS) was implemented into healthcare at the Karolinska University Hospital for diagnosis of rare diseases across a broad range of disease areas. Up to 2019, more than 3000 patients were analyzed resulting in specific molecular diagnoses for >1200 patients. This was achieved through a close collaboration between the hospital and the Clinical Genomics facility at Science for Life Laboratory, developing bioinformatic tools and workflows and establishing in a collaborative community where multidisciplinary teams focus on different disease groups and share genome data. Inborn Errors of Metabolism was the starting point, providing a proof-of-concept where WGS data are integrated with highly specialized laboratory medicine and multidisciplinary clinical expertise, enabling rapid interpretation of data and direct translation into individual patient management. The work has enabled introduction of WGS as a confirmatory test in the national Swedish newborn screening program, resulting in a higher yield of genetic diagnoses compared to conventional genetic testing.

I02. **The Impact of the Second-Tier Tests and Role of NGS on NBS Performance—Could They Become First Line Tests?**

Belén Pérez

Centro de Diagnóstico de Enfermedades Moleculares, Centro de Biología Molecular, Universidad Autónoma de Madrid, CIBERER, IdiPAZ, Madrid, Spain

The main purpose of newborn screening (NBS) programs is to diagnose genetic disorders early, allowing treatment to begin before symptoms appear. Inborn errors of metabolism (IEM) make up a phenotypically and genetically heterogeneous group of rare disorders resulting from defects in certain metabolic pathways, which in many cases cause the accumulation of toxic intermediate metabolites. To date, more than 1500 different IEM were identified. In the last decade, the use of tandem mass spectrometry (MS/MS) in expanded NBS for IEM has become mandatory in Western countries, such as Spain. The quantification of amino acids and acylcarnitines in dried blood spots (DBS) by MS/MS allows the simultaneous detection of more than 30 metabolic disorders, including those associated with amino acid, organic acid, and fatty acid metabolism. When a metabolic abnormality is detected in DBS analysis, other samples (plasma and urine) are collected for confirmatory biochemical testing—commonly for amino acids, homocysteine, acylcarnitines, and organic acids. In many cases, this may complete a differential diagnosis since certain biomarkers are related to defects in different genes (locus-heterogeneous disorders). The present work describes the value of genetic analysis as a confirmatory measure following the detection of suspected IEM in the Spanish newborn mass spectrometry screening program. DNA samples extracted form DBS samples were analyzed by next-generation sequencing (customized panel, clinical exome, whole exome or transcriptional studies). We were able to identify positive cases, carriers, false positive and cases with diallelic inheritance. Carriers and false positive were mainly identified in VLCADD. The identification of a specific variants can reveal the need for a specific treatment such as the administration of tetrahydrobiopterin (BH4) in phenylketonuria (PKU), or vitamin B12 in some cobalamin disorders. In addition, disease photocopies were resolved by genetic analysis and new genes associated with diseases were also identified. This also expanded our knowledge of classic phenotypic characteristics and provide insight into associations between “old diseases” and “new genes”. The results open the opportunity to a new extension of the neonatal screening program to other actionable IEMs by genetic biomarkers

I03. **Second Tier Genetic Testing in the Norwegian National Screening Program**

Janne Strand

Oslo University Hospital, Department of Newborn Screening, Oslo, Norway

In 2012, Norwegian Newborn Screening expanded from two to 23 disorders, introducing genetic testing methods to our screening program. The legal basis for this expansion allows us to test genetically for the disorders in the program without the need for written consent. Since 2012, we added three more disorders giving a total of 26, including SCID and SMA which rely heavily on genetic methods for precise results.

Cystic fibrosis was the first disorder to benefit from the combination of biochemical and genetic testing, and the first disorder to adopt next-generation sequencing (NGS) (in 2015). Since 2012, 2nd-tier genetic testing has gradually been introduced for the majority of disorders, supporting decision-making and increasing the specificity and sensitivity of our screening. This has allowed us to lower 1st-tier cutoffs to avoid false negatives, while simultaneously excluding many false positives.

The introduction of SCID screening paved the way for the use of NGS gene panels in our newborn screening program. NGS is a cost-effective and efficient way of evaluating many genes simultaneously, and we have created a panel consisting of all NBS relevant genes that enables us to streamline our genetic analyses into a single approach applicable to most screening conditions with a turnaround time of 2–3 days. 

Genetic testing has in combination with 2nd-tier MS analysis contributed to an increase in the positive predictive value from 26% in 2012 to around 80% in recent years. Despite the advantages, there are also shortcomings to this technology, and relying on genetics alone is not a viable option. Biochemistry in combination with rapid confirmatory genetics provides a reliable high precision screening approach that enables us to find the children that should be found, without unnecessarily distressing the families of healthy children. 

I04. **Maternal Vitamin B12 Deficiency: Definition in Relation to Newborn Screening, Are These True Positives?**

Anne-Lise Bjørke-Monsen ^1,2^

^1^ Department of Medical Biochemistry and Pharmacology, Haukeland University Hospital, Bergen, Norway

^2^ Laboratory of Medical Biochemistry, Innlandet Hospital Trust, 2609 Lillehammer, Norway

The cobalamin status of a neonate depends on maternal cobalamin stores during pregnancy, placental function and gestational age at birth. After birth, maternal cobalamin status will continue to have an impact, as long as the infant is exclusively breastfed. As an adequate cobalamin status is crucial for normal development of the central nervous system, securing an optimal maternal cobalamin status is important. Pregnancy, lactation and infancy are life stages characterized by increased needs and risk of deficiency of many micronutrients. Cobalamin deficiency in both mothers and infants have been reported from countries all over the world, but the prevalence varies. This might be due to local dietary factors, but also to differences in the decision limits defining cobalamin deficiency.

Cobalamin deficiency is associated with the subtle clinical symptoms, particularly in young infants, and may be difficult to detect. Cobalamin status can be assessed by serum cobalamin alone or in combination with the metabolic markers plasma total homocysteine (tHcy) and/or metylmalonic acid (MMA). Currently there is no international consensus on decision limits for cobalamin deficiency, but serum cobalamin concentrations < 148 pmol/L, plasma tHcy > 15 µmol/L and plasma MMA > 0.37 µmol/L are commonly used in adults. However, the metabolic markers start to increase already when serum cobalamin falls below 550 pmol/L, indicating biochemical insufficiency and the concentrations increase sharply below 250–300 pmol/L, indicating intracellular cobalamin deficiency, in both children and adults. 

In pregnancy, lactation and infancy, profound physiologic changes have an impact on the concentrations of serum cobalamin, plasma tHcy and MMA and the relation between them. Decision limits for older children and adults cannot be used, something which hamper evaluation of micronutrient status in these life stages.

Maternal serum cobalamin concentrations progressively decrease during pregnancy and increase up to 40% six weeks postpartum. The concentrations of both plasma tHcy and MMA are substantially lower in pregnant women, but increase from week 18 of pregnancy to six weeks postpartum: median plasma tHcy 3.9 to 7.7 μmol/L (*p <* 0.001), and MMA 0.13 to 0.17 μmol/L (*p <* 0.001).

During the first months of life, serum cobalamin concentrations decrease, while plasma tHcy and MMA increase. The lowest cobalamin levels and highest tHcy and MMA levels seen in exclusively breastfed infants aged 4 to 6 months. Plasma tHcy ≥ 6.5 µmol/L, representing the 97.5 percentile in infants given a single intramuscular dose of 400 µg hydroxycobalamin, has been suggested as a decision limit for cobalamin deficiency in infants. This supplementation reduced median plasma tHcy by 39% and MMA by 66%, indicating that high concentrations of the metabolic markers in infants, do indeed reflect cobalamin insufficiency and not organ immaturity. A 400 µg hydroxycobalamin intramuscular dose also improved motor function in young infants with a biochemical profile indicative of moderate cobalamin deficiency, implying that an optimal cobalamin status is important for a rapidly developing nervous system. Maternal serum cobalamin < 275 pmol/L (measured by immunoassay) in pregnancy week 18 has been shown to be associated with an increased risk (OR 4.2 (95% CI 1.5, 11.5) of infant cobalamin deficiency at six months (defined as tHcy ≥ 6.5 μmol/L). 

A biochemical profile indicative of a moderate impaired cobalamin function is observed in more than two thirds of mainly breastfed young Norwegian infants below six months. As we know that also moderately low cobalamin status may hamper neurodevelopment in infants, this finding has clinical implications. Current newborn cobalamin screening may be able to identify the severely deficient infants, but without optimal decision limits, we may fail to detect the majority of cobalamin deficient neonates.

I05. **Maternal Vitamin B12 Deficiency—Impact on Screening for MMA/PA**

Raquel Yahyaoui

Málaga Regional University Hospital, Málaga, Spain

When undiagnosed, infant vitamin B_12_ deficiency can result in anemia, failure to thrive, developmental delay or regression, and cerebral atrophy. It is most commonly caused by maternal vitamin B_12_ deficiency. Biochemically, vitamin B_12_ deficiency leads to an accumulation of total homocysteine (tHcy), methylmalonic acid (MMA), and propionylcarnitine (C3). Although vitamin B_12_ deficiency is not considered a primary target of expanded newborn screening (NBS) programs, markers for methylmalonic and propionic acidemias (elevated C3 and C3/C2) may identify vitamin B_12_-deficient newborns. The sensitivity of these markers for vitamin B_12_ deficiency in NBS is still unknown, but a previous study of our group showed that markers C3, C3/C2 and C3/C16 exhibit a negative correlation with maternal levels of vitamin B_12_ in the first trimester of pregnancy. Some NBS programs also flag low methionine levels.

Causes of maternal vitamin B_12_ deficiency include adherence to a diet that excludes or has limited amounts of animal products, pernicious anemia, gastric surgeries and others. Unrecognized neonatal vitamin B_12_ deficiency worsens if the infant is breastfed without vitamin B_12_ supplementation. Clinical presentation of vitamin B_12_ deficiency is often nonspecific which can lead to a delay in diagnosis and treatment. Irreversible neurologic damage results from prolonged vitamin B_12_ deficiency; however, the extent and degree of disability depends on the severity and duration of the deficiency. 

To perform vitamin B_12_ deficiency NBS, most of the laboratories verify the persistence of the alteration of these markers in a DBS second specimen before performing the diagnostic study, what implies a delay in diagnosis and a considerable false positive rate. In recent years, many laboratories have implemented a second-tier test (tHcy and MMA by LC-MS/MS) that significantly improves sensitivity and specificity, which is why many consider it a mandatory test.

There is great heterogeneity in the tests performed in the diagnostic confirmation study, but it is advisable to study both the newborn and the mother, as well as to identify the origin of the deficiency. In our center, all cases with persistently high levels of C3 and/or C3/C2 are studied further, both mother and child, evaluating CBC, acylcarnitines, tHcy and vitamin B_12_ levels in plasma, and organic acids in urine samples. Mothers are also tested for gastric parietal cells (GPC) and intrinsic factor (IF) serum antibodies.

Vitamin B_12_ deficiency detected through NBS presents with a high frequency (1:5335 in our population). In our experience, the ratio C3/C2 is a more sensitive marker than C3 for the detection of vitamin B_12_ deficiency. Most newborns are exclusively breastfed at diagnosis. Many cases of probable maternal pernicious anemia are usually detected (35% of the cases) and newborns of mothers with pernicious anemia had a significantly more severe deficiency than the rest of newborns. If the deficiency is present in the newborn but not in the mother, a genetic defect in the absorption, transport, or intracellular metabolism of B_12_ vitamin is investigated. All confirmed cases (infants and mothers) are treated with oral or intramuscular vitamin B_12_ and do not present hematologic or neurological symptoms during follow-up.

In summary, identification of newborns with nutritional vitamin B_12_ deficiency is an additional benefit of NBS programs. The inclusion of the ratio C3/C2 as a primary marker and the implementation of second-tier testing increase the program’s sensitivity and specificity. If a vitamin B_12_ deficiency is suspected, then both the mother and the infant should be promptly evaluated. Maternal screening for pernicious anemia is cost-effective as it may identify the cause in a significant percentage of cases, which are also the most serious. NBS programs should consider infants detected with confirmed vitamin B_12_ deficiency to be true-positive cases.

I06. **Maternal Iodine—Too Little and Too Much**

Natasha Heather

Newborn Screening, LabPlus, Auckland, New Zealand

Iodine is an essential trace element required for the production of thyroid hormones. Iodine deficiency is the world’s leading cause of preventable brain damage, with fetal brain development vulnerable to even mild-moderate deficiency in the first trimester. Around the time of birth, maternal iodine deficiency or excess can both impact newborn screening TSH concentrations and lead to transient hypothyroidism in the newborn. Practical examples from the newborn screening laboratory will be described.

I07. **If You Can Screen for 300 Disorders Why Stop at 30?**

Martina Cornel

Amsterdam University Medical Centres, The Netherlands

Criteria to include conditions in new-born screening (NBS) programmes differ between jurisdictions. As for any screening, the harm of screening should not outweigh the benefits. Jurisdictions therefore have procedures to evaluate pros and cons, based on scientific evidence as well as views of key stakeholders. Often such procedures evaluate each potential target condition. Meanwhile technologies develop in a different way: one test can identify many conditions. This is true for tandem mass spectrometry, genome sequencing techniques, pulse oximetry, etc.

From a patient or parent’s perspective, having a child with a very rare condition that is diagnosed after a long diagnostic odyssey is a tragedy. If harm can be avoided, either by treatment or by limiting the number of unnecessary diagnostic procedures, limiting uncertainty and unfavorable psychological effects, these all could count in the evaluation of pros and cons. As long as the interest of the infant is not harmed by early diagnosis, the advantages for parents, family, and society could be included in the evaluation.

The differences between countries in target conditions are explained by a multitude of reasons. Only the analysis of dried blood spots was included, and pulse oximetry to evaluate critical congenital heart disease or other reasons for low oxygen levels was overlooked. In a focus on inborn errors of metabolism, vitamin B12 deficiency was not included. Conditions that lack scientific evidence for the benefit of early diagnosis and treatment account for the majority of differences. We could define the collection of scientific evidence on rare disorders as a secondary goal of NBS, but so far the public health perspective started from scientific evidence of benefits for the new-born. A technology driven approach would allow for the inclusion of hundreds or thousands of disorders, but clearly the potential harms of overdiagnosis and false positives should be limited. 

In this debate we will discuss two sides of the coin with an intention to share views internationally. 

I08. **Just Because You Can Doesn’t Mean You Should—Screen Newborns for Any Disorder**

Wybo Dondorp

Department of Health Ethics & Society, Maastricht University, Maastricht, The Netherlands

Usually screening aims to achieve health gains for those tested, and this of course is also how the classical aim of NBS is understood: benefiting the child through the timely identification of serious but treatable disorders. However, what if with the same test we can screen for a much wider range of conditions? 

There are at least three possible perspectives to address this question. One is the traditional framework of responsible screening as developed by Wilson and Jungner and successors. A second is that of using available data to maximize health benefits not just for individuals, but also for families and future patients. A third is that of a presumed parental right to have their child tested for whatever condition they find important. 

I will argue that there are good reasons to stick to the traditional framework. Not just because of concerns about undermining neonatal screening as a highly successful public health service, but also for the more principled ethical reason of not reducing children to mere means. While it does not follow from this that wider screening should rejected, it does entail that any proposals should be assessed in terms of evidence of a positive benefit to harm ratio for the child. This allows counting in benefits for the child that are psychosocial rather than strictly medical, or based on a broader understanding of treatability than only in curative terms. Family benefits may count as well as long as they also benefit the child. However, counting in third-party benefits becomes problematic when they are presented as separately outweighing any possible harms to the child.

I09. **Ethical Dilemmas with the Identification of Conditions of Unclear Significance**

Trine Tangeraas

Oslo Univ Hospital, Department of Newborn Screening, Oslo, Norway

In the era of expanded newborn screening (NBS), a substantial proportion of attenuated phenotypes of CF, VLCADD, MCADD, CTD and partial biotinidase deficiency are detected. This talk will focus on the challenges that accompanies the findings of unequivocal clinical significance using the experience from the Norwegian NBS program as an example. Collaborative effort is required to develop adjusted guidelines for treatment (if any) and follow-up (if needed) of milder phenotypes to mitigate the burden on the family and health care system.

I10. **European Overview**

Dimitris Platis

Institute of Child Health, Athens, Greece

Neonatal Screening (NBS) was first introduced during the 1960′s with the screening of phenylketonuria. Subsequent technological advances, such as the introduction of tandem mass spectrometry in the late 1990′s which allowed for the simultaneous determination of multiple analytes made it possible to screen for 40–50 conditions from a single blood spot. Lately, advances in the field of molecular diagnostics significantly assisted in the addition of several additional disorders (cystic fibrosis, severe combined immunodeficiency and spinal muscular atrophy). Therefore, the developments in the field of Neonatal Screening are constant, produce challenges and need to be recorded. 

The achievements over the last decade in Europe based on a recent ISNS survey and collected data from 51 European countries will be highlighted making it possible to identify areas where further progress can be made, therefore enabling the exchange of knowledge among neighboring countries in an effort to strengthen collaboration in Europe. 

I11. **IT Infrastructure for Long-Term Follow-Up for Newborn Screening in Sweden**

Rolf Zetterström and Lene Sörensen

Centre for Inherited Metabolic Diseases, Karolinska University Hospital, Stockholm, Sweden 

After the extension of the Swedish newborn screening program in 2010 from five to 24 disorders, it became clear that a structured program for following up the long-term outcome for children was missing. In 2011, there was a national push for registries in Sweden and we received funding to develop a registry for inherited metabolic diseases (“registret för medfödda metabola sjukdomar”, RMMS). In 2013, we registered the first patient and today more than 35 disorders, including all 22 metabolic disorders in the national screening program, are included. Since 2018 all blood phenylalanine results from Swedish PKU patient follow-up tests are electronically uploaded and from two specialist laboratories other patients control measures such as plasma amino acids and organic acids are also directly exported to RMMS. Patient reported outcome measures such as DISABKIDS and PKU-QoL are easily accessible for both professionals and patients and several psychometric tests such as the Wechsler intelligence scales also included have proven very useful for neurocognitive assessment. In conclusion, the Swedish registry is now a powerful system allowing long-term follow-up of patients. Direct export of data from the laboratories into the registry, as well as easy data access for professionals and patients and families, have greatly helped in making the registry user friendly.

I12. **The Value of European Registries and Networks for Changing Practice and Informing Guidelines**

Stefan Kölker

Division of Metabolic Medicine and Child Neurology, Center for Child and Adolescent Medicine, Heidelberg University Hospital, Heidelberg, Germany

Although patient registries are considered to be essential for epidemiological and clinical research for rare diseases, enabling achieving a sufficient sample size, long-term observational studies of newborn screening (NBS) cohorts are still the neglected part of NBS programs worldwide. This is a major shortcoming for accurately assessing the impact of NBS on long-term health outcomes of screened individuals as well as evaluating the health economical and societal benefits of NBS programs.

Patient registries allow describing clinical outcomes as well as the efficacy and safety of the health impacts of current diagnostic and therapeutic interventions used under real-world conditions, aiming to study a broad range of individuals with a broad phenotypic spectrum and to achieve generalizable results. This approach has enormous potential for the evaluation and optimization of NBS programs, as well as for the harmonization of evidence-based recommendations:

1. A solid understanding of phenotypic diversity is indispensable to compare the case mixes of clinical phenotypes in newborn screening cohorts to the pre-screening era.
2. This helps to identify new groups of individuals with attenuated or potentially benign phenotypes and to improve case definitions by establishing early prediction models for clinical severity and by establishing second or multiple tier strategies for NBS.
3. Unraveling phenotypic diversity also guides therapeutic decision-making and helps to reduce the risk of over- and under-treatment, paving the way to personalized medicine.
4. Finally, evidence gathered from large and prospectively followed cohorts informs guideline development and harmonizes diagnostic and therapeutic strategies.

This presentation will provide examples from the long-term observational study of the German newborn screening cohort as well as from European patient registries and consortia such as E-IMD and U-IMD to support this notion. 

I13. **Value of Late Treatment of PKU Patients Born before Newborn Screening**

Maria Giżewska

Department of Paediatrics, Endocrinology, Diabetology, Metabolic Diseases and Cardiology of the Developmental Age, Pomeranian Medical University in Szczecin, Szczecin, Poland

Despite over 60 years of newborn screening for phenylketonuria (PKU), there are still regions of the world where the procedure was started much later or it is not performed at all. 

Late-diagnosed or untreated PKU patients can be found in different environments including patients’ families, social welfare homes or communities of migrants coming from countries where there is no efficient newborn screening program. It is important to identify these patients as specific therapeutic intervention with a phenylalanine-restricted diet may be beneficial for them. If untreated, hyperphenylalaninemia leads to severe multidirectional brain damage and results in intellectual disability, speech difficulties, epilepsy, microcephaly and neurological impairment.

Up to the age of several years, late-treated children with PKU can benefit from treatment and are able to catch up intellectually therefore, it is recommended to start therapy in all of them. In some of older untreated patients, especially adults, even late introduction of treatment can lead to significant improvement in quality of life and facilitation of every-day care. Patients become calmer exhibit decrease in irritability, hyperactivity and aggressive behavior. In some of them neurological symptoms decrease—severity of epilepsy reduces and intellectual functioning improves. As it is hard to predict who will respond to dietary treatment, an introduction of dietary intervention is this particular group of patients is worth considering.

I14. **Newborn Screening for Spinal Muscular Atrophy, SMA: Achievements, Challenges and Controversies**

Jan Kirschner

Department of Neuropediatrics, University Hospital Bonn, Germany

Spinal muscular atrophy (SMA) is a neurodegenerative disease with a birth prevalence of about 1:10,000 caused by autosomal recessive mutations in the *SMN1* gene. The disease is associated with a broad spectrum of disease severity and the main predictor of severity is the number of copies of the *SMN2* gene. The most common subtype is SMA type 1, which is associated with disease onset during the first six months of life and early mortality due to respiratory failure. New treatment options including antisense oligonucleotides, small molecules and gene therapy have a significant impact on the disease course. Presymptomatic initiation of treatment often facilitates almost normal motor development. Therefore, an increasing number of newborn screening (NBS) programs include SMA as a target disease. Most programs screen for homozygous deletions by PCR from dried blood spots but not for carrier status. Pilot projects have already shown that NBS can significantly improve the outcome of SMA, but it is also associated with new challenges. It is not yet known if all affected individuals should be treated immediately or if a watch-and-wait strategy should be followed for patients with a higher number of *SMN2* copies predictive of a later onset phenotype. The costs for drug treatment are extremely high and access to treatment in a timely manner can be challenging. In addition, after diagnosing SMA, parents with an apparently healthy newborn have to take decisions about invasive treatments, such as repetitive lumbar punctures for administration of antisense oligonucleotides or gene therapy.

I15. **SMA—Survey of the European Screening Laboratory Experience**

François Boemer

Centre Hospitalier Universitaire de Liège, Belgium

Development and reimbursement by health care systems of new treatments for SMA have prompted the implementation of newborn screening (NBS) programs in different countries. The first published pilot studies were carried out in Taiwan and in New York State in 2017. Subsequently, several countries or regions have initiated trials to implement NBS programs. We will review the different corresponding initiatives in Europe, focusing on screening organization, consent requirement, analytical approaches, and follow-up of positive results.

I16. **A European Overview of Newborn Screening for SCID Experiences and Future Perspectives**

Ana Argudo Ramirez

Hospital Clinic Barceona, Spain

Severe combined immunodeficiency (SCID), the most severe form of T-cell primary immunodeficiency (PID), includes a group of inherited defects characterized by severe T-cell lymphopenia (TCL). Patients with SCID require prompt clinical intervention to prevent severe life-threatening infections. Early detection of this disease through neonatal screening has been shown to improve the SCID infants’ survival mainly by performing the hematopoietic stem cell transplant (HSCT) prior to the onset of infectious complications. T-cell receptor excision circle (TREC) is the biomarker which detects a low- or non-naive T-cell number and can be used in a cost-effective way to screen for SCID with high sensitivity. Different strategies are being used in Europe: TREC, TREC/KREC, Ado and dAdo metabolites for adenosin deaminase deficiency (ADA-SCID), even introducing a second-tier test (NGS). Psychological support for the parent is an issue that is also gaining importance. 

In Europe, SCID is routinely screened for in 12 countries or regions: Andorra (2018), Denmark (2020), Finland (2019), Germany (2019), Iceland, Italy (Tuscany), Netherlands (2021), Norway (2018), Poland (regional, 2017), Spain (Catalonia, 2017), Sweden (2019) and Switzerland (2019). In other countries or regions pilot projects were, are, or will be running: France (2015–2017), Ireland (2021), Slovakia, Spain (Seville, 2014–2015) and United Kingdom (Sept 2021).

As in the USA, European results show that after more than 1,250,000 newborns were screened, no classical SCID was missed by screening programs so far. Infants with non-SCID TCL were also identified, diagnosed, and managed, although European NBS programs should try to unify how to classify these cases in order to compare the results obtained. NBS for SCID saves lives, is cost-effective, and it has to continue to be included in all regions and countries in Europe. 

I17. **The Dutch Newborn Screening Pilot Program for X-Linked Adrenoleukodystrophy (ALD)**

Stephan Kemp

Amsterdam University Medical Center, The Netherlands

X-linked adrenoleukodystrophy (ALD) is a devastating metabolic disorder affecting the adrenal glands, brain, and spinal cord. Males with ALD are at high risk of developing adrenal insufficiency or progressive cerebral white matter lesions (cerebral ALD) at an early age. If untreated, cerebral ALD is often fatal. Women with ALD are not at risk of adrenal insufficiency or cerebral ALD. Newborn screening for ALD in males enables prospective monitoring and timely therapeutic intervention, therefore preventing irreparable damage and saving lives. The Dutch Ministry of Health adopted the advice of the Dutch Health Council to add a boys-only screen for ALD to the newborn screening panel. The recommendation made by the Dutch Health Council to only screen boys, without gathering any unsolicited findings, posed a challenge. We were invited to set up a prospective pilot study that became known as the SCAN study (SCreening for ALD in the Netherlands). The objectives of the SCAN study are: (1) designing a boys-only screening algorithm that identifies males with ALD and without unsolicited findings; (2) integrating this algorithm into the structure of the Dutch newborn screening program without harming the current newborn screening; (3) assessing the practical and ethical implications of screening only boys for ALD; and (4) setting up a comprehensive follow-up that is both patient- and parent-friendly. We successfully developed and validated a screening algorithm that can be integrated into the Dutch newborn screening program. The core of this algorithm is the “X-counter.” The X-counter determines the number of X chromosomes without assessing the presence of a Y chromosome. The X-counter is integrated as second tier in our four-tier screening algorithm. Furthermore, we ensured that our screening algorithm does not result in unsolicited findings. Finally, we developed a patient- and parent-friendly, multidisciplinary, centralized follow-up protocol. Our boys-only ALD screening algorithm offers a solution for countries that encounter similar ethical considerations, for ALD as well as for other X-linked diseases. For ALD, this alternative boys-only screening algorithm may result in a more rapid inclusion of ALD in newborn screening programs worldwide.

I18. **Newborn Screening for Lysosomal Storage Diseases, LSDs—The Italian Experience**

Giancarlo la Marca ^1,2^

^1^ Department of Experimental and Clinical Biomedical Sciences, University of Florence

^2^ Newborn Screening, Clinical Chemistry and Pharmacology Laboratory, Meyer Children’s Hospital, Florence, Italy

The spread of national newborn screening (NBS) programs has provided significant benefits in the diagnosis and early treatment of several rare, heritable conditions, preventing adverse health outcomes for most affected infants. New technological developments have enabled the implementation of testing panel covering over 50 disorders. Moreover, the availability of treatments and the importance of early intervention have stimulated some Italian Regions to extend their panel to some Lysosomal Storage Disorders (LSDs) with the aim of early intervention in preventing irreversible impairment or severe disability. Here we report the results of newborn screening for Mucopolysaccharidosis type I (MPS-I), Pompe, Fabry, and Gaucher diseases in North East and Central Italy in the period 2014–2021. Activities of acid β-glucocerebrosidase (Gaucher), acid α-glucosidase (Pompe), acid α-galactosidase (Fabry), and acid α-L-iduronidase (MPS-I) in dried blood spots (DBS) were quantified by tandem mass spectrometry (MS/MS). From January 2014 to June 2021, 328,970 newborns were screened for the four LSDs. Confirmatory testing certified low activity and pathogenic gene variants and/or VUS (pseudodeficiencies were not included): 43 Pompe, 12 Gaucher (only in North East), 34 Fabry, and 2 MPS-I. The overall incidence of confirmed positive newborns screened for Pompe disease was 1/7650, Gaucher 1/27,400, Fabry 1/9675 and MPS-I the rarest, 1/164,385. The high number of confirmed positive newborns and the availability of established and emerging therapies could support a European common discussion to extend the current newborn screening panel to these LSDs.

I19. **What Happens after Recall, Who Should Give the Results to Families?**

Trine Tangeraas

Oslo University Hospital, Department of Newborn Screening, Oslo, Norway

The reporting of a presumptive diagnosis from newborn screening (NBS) to the families differs between countries and even regions depending on the organization of the NBS programs, health care systems, geographical aspects, and traditions. Additionally within the specific screening program, the process of information may vary according to the screening condition. Ideally a health care professional with knowledge of the screening process and with clinical disease expertise should communicate the screening results. The family should be told both in a structured manner and in a prompt face-to-face meeting, no matter whether the result is a true positive or false positive case, but this is not always feasible. This talk will present the practice of the Norwegian NBS program as a starting point to a broader discussion on approaches to optimize the interactions with the family. 

I20. **The Maternal Stress of False Positive Newborn Screening Results**

Vera Franková, Hermánková, R., Dohnalová, A., Dragomirecká, E., Pešková, K., Oliveriusová, P., Votava, F., Holubová, A. and Kožich, V.

Charles University, Prague, Czech Republic

Background: Newborn screening (NBS) programs continuously expand in many countries. The inclusion of additional disorders leads to an increased occurrence of false positive (FP) results that usually cause distress for parents until the results are evaluated as negative. The purpose of this study was to evaluate parental stress and perceptions of child’s health in mothers of children with FP result after longer term.

Methods: We mailed a survey to 401 mothers of children with a false positive (FP) and to 810 mothers with a negative result six months after the completion of NBS. The survey contained questions about demographic data, healthcare use, understanding of the result, and concerns about the child’s health. To measure stress in parent-child relationship Parenting Stress Index Short Form (PSI-SF) was also mailed to both groups of mothers. 

Results: The overall response rates in the group of mothers of children with a FP result and the control group were 23% and 26%, respectively. After excluding incomplete responses we analyzed data from 75 mothers from the FP group and 201 from the control group. Education was the only demographic factor in which these two groups differed significantly (39% vs. 55% of mothers with university degree; *p* < 0.05). There was no difference between the groups either in the overall stress score or in the parental distress, difficult child and parent-child subscales of PSI-SF. Twenty-eight percent of mothers from the FP group stated they had still concerns about the disorder in their children, but there was no significant difference in the number of hospitalizations or frequency of common childhood diseases when compared to control group. 

Conclusions: A false positive result may lead to immediate distress and anxiety when communicated to mothers. Although concerns about the disease persist in some mothers, they do not lead to an increased use of healthcare services and do not influence the stress in parent-child relationship six months after completion of NBS. 

This study was supported by projects PROGRES Q26 from Charles University and RVO VFN 64165. 

I21. **Dutch Parents’ and Professionals’ Perspectives on Expanding Neonatal Bloodspot Screening to Newer Disorders**

Lidewij Henneman 

Amsterdam University Medical Center, The Netherlands

The number of conditions included in Neonatal bloodspot screening (NBS) is expanding through technological and therapeutic developments, which can result in health gain for more newborns. NBS expansion, however, also poses healthcare, ethical, and societal challenges. The PANDA study (**P**sychosocial **A**spects of **N**ewborn Screening for **D**isorders **A**ssessed) anticipates current developments in NBS in the Netherlands. In 2021, the Dutch screening program was expanded to 25 disorders, based on recommendations from the Health Council of the Netherlands. The expansion is still ongoing with many challenges still ahead. The aim of the PANDA study is to investigate a multi-stakeholders’ perspective on the expansion of the Dutch NBS program. A mixed methods design was used including semi-structured interviews conducted with professionals, including healthcare professionals, test developers, and policy makers, and parents of children with normal and abnormal NBS results. Addressed themes were: (1) Benefits and challenges of current expansion, (2) Expectations regarding future developments, and (3) NBS acceptance and consent procedures. Moreover, a large survey study was performed among parents who participated in NBS. Findings of interviews and survey will be presented. This study aims to fuel public debate and contribute to future policy decisions and shaping NBS practices.

Funding: Netherlands Organization for Health Research and Development (grant no. 543,002,006).

## 2. Oral Presentations

O01. **Application of a Protocol for Structured Follow-Up and Texting of Inadequate or Bordeline-Positive Screen Results**

Natasha Heather, Detlef Knoll, Keith Shore, Mark de Hora, Dianne Webster and Lisa Morgan

Newborn Screening, LabPlus, Auckland, New Zealand

Background: According to the New Zealand newborn metabolic screening program monitoring parameters, second samples should be received in the screening laboratory within 10 days of the request. Second samples are requested when the initial sample is inadequate (e.g., too early, poor quality samples) or the result is borderline-positive. In 2015 a national protocol for structured follow-up and texting or phoning second sample requests was introduced.

Aim: To evaluate whether use of a structured follow-up protocol improved the completion rate and timeliness of receipt of second cards.

Method: Under the structured protocol all second sample requests were immediately telephoned or texted to the lead maternity carer, in addition to the standard written report issued. Text or phone reminders were sent at 1, 2, 3, and 4 weeks or until the sample was received. If the second sample was still pending, the formal report was re-issued at 3 weeks and task closed at 4 weeks. National data on second card requests was monitored following implementation of the structured follow-up protocol.

Results: The completion rate for receipt of second cards increased from 92.5% of 1372 requests in 2016 to 97.2% of 831 requests in 2019. Reasons for incomplete follow-up included parental decline and task closure due to loss of follow-up. The proportion of second samples received within 10 days of request improved from 67% in 2016 to 77% in 2019.

Conclusion: A structured follow-up protocol that included text or phone contact in addition to a formal written report led to earlier and more complete receipt of second samples. This is likely due to practitioners receiving the request more quickly and to the laboratory adopting a consistent approach to repeated reminders. Texting is a useful adjunctive method for screening programs to communicate with individual practitioners.

O02. **An Introduction to the Screen4Rare Stakeholder Platform for Equity in Neonatal Screening in Europe**

Johan Prevot ^1^, Gerard Loeber ^2^, Martine Pergent ^1,3^, Isabelle Meyts ^4^, Nizar Mahlaoui ^1,5^, Elliot Tricot O’Farell ^6^, Yordan Aleksandrov ^6^, Leire Solis ^1^, Peter CJI Schielen ^2^ and James Bonham ^2^

^1^ International Patient Organisation for Primary Immunodeficiencies (IPOPI), Brussels, Belgium

^2^ International Society for Neonatal Screening (ISNS), Stichtse Vecht, The Netherlands

^3^ IRIS-French Patient Organization for Primary Immunodeficiencies

^4^ European Society for Immuno Deficiencies (ESID), Delft, The Netherlands

^5^ CEREDIH (Centre de Référence Déficits Immunitaires Héréditaires) & Unité d’Immuno-Hématologie & Rhumatologie pédiatrique, Hôpital Universitaire Necker-Enfants Malades, Assistance Publique-Hôpitaux de Paris (AP-HP), Paris, France

^6^ RPP Group, Brussels, Belgium

The ISNS has been involved in efforts to promote greater equity of neonatal screening provision in Europe, leading to the establishment of Screen4Rare.

Here, we introduce the founding organizations of Screen4Rare, its history, aims, key achievements and future.

Initial ideas that led to Screen4Rare were exchanged at a meeting in 2015 where IPOPI (International Patient Organisation for Primary ImmunoDeficiencies) made the case for a European policy on newborn screening (NBS). ISNS and IPOPI joined forces, later also with the European Society for Immunodeficiencies (ESID) and between 2015–2018, supported by RPP group (an advisory organization specialized in EU public health), collaboration intensified and connection with EU public health representatives was sought. A meeting at DG Health in 2020 initiated the establishment in May 2020 of Screen4Rare, as a stakeholder platform hosted by the EU Health Policy Platform (EU-HPP).

Screen4Rare is now a multi-stakeholder initiative launched by IPOPI, ISNS and ESID aimed at exchanging knowledge and best practices on NBS for rare diseases for equitable access to good quality NBS.

December 2020, Screen4Rare presented a ‘Call to Action’ (supported by 30 EU-MEPs), to (1) find support for generally accepted overarching guidelines, (2) build a platform for stakeholders and (3) try to position the EU as a point for data collection on rare diseases newborn screening. In 2021, Screen4Rare was instrumental in the launch of International Neonatal Screening Day on 28 June 2021.

Screen4Rare is building relations with European partners: the European Reference Networks, Public Health representatives of member states and the European commission, MEPs and international patient organizations.

Future aims include expanding the network and delivering on the call to action goals.

O03. **Enhancing Data-Driven Disease Detection in Newborns: A National Data Platform for Modernizing Newborn Screening Data Analytics and Interpretation**

Amy Gaviglio and Carla Cuthbert

CDC, Newborn Screening and Molecular Biology Branch, Atlanta, GA, USA

ED3N (pronounced “Eden”) is a National Newborn Screening Data Platform being developed by the Division of Laboratory Sciences Newborn Screening and Molecular Biology Branch (NSMBB).

The newborn screening system is facing an increase in data analytic challenges associated with ongoing expansion of the number of newborn screening diseases and the increased complexity of correlating biomarkers with disease risk and severity.

Through continuous collaboration between newborn screening programs and NSMBB, ED3N can assist programs by securely collecting, processing, and analyzing demographic, biochemical, molecular, and clinical data in near real-time. Ultimately, the goal of ED3N is to aid programs in assessing risk of disease at the time of screening. The presentation will discuss the overarching infrastructure and planned deployment of ED3N, which uses an interconnected modular approach as outlined below:

Biochemical module:

- Development and use of laboratory testing harmonization techniques to allow for aggregation of data across various platforms
- Use of machine learning for predictive algorithms to improve risk assessment Molecular module
- Development of streamlined bioinformatics pipeline with structured variant interpretation capabilities
- Newborn screening-specific workflows and genotype sharing across programs
- Linkage to biochemical and clinical module for analysis of genotype-phenotype relationships
- Clinical module
- Use of standard case definitions
- Collection of diagnostic data from both NBS programs and specialists

The newborn screening system generates an immense amount of data that is housed in programmatic data silos—without interoperability and aggregation, we cannot leverage the power of connected data for improving disease detection and understanding.

O04. **Global Impact of COVID-19 on Newborn Screening Programs**

Urh Groselj, Vanesa Koracin, J. Gerard Loeber, Matej Mlinaric, Tadej Battelino, James R. Bonham and COVID-NBS ISNS Global Network

University Children’s Hospital Ljubljana, University Medical Centre Ljubljana, Faculty of Medicine, University of Ljubljana, Department of Endocrinology, Diabetes and Metabolic Diseases, Ljubljana, Slovenia

A global pandemic of coronavirus disease 2019 (COVID-19) has presented extraordinary disruption to healthcare services and exposed them to numerous challenges. Newborn screening (NBS) programs were also affected, but scarce data exist on the impact of COVID-19 on NBS.

We conducted an international survey to assess the global impact of COVID-19 on NBS with the main aim to gather experiences of the COVID-19 pandemic from a large and representative number of NBS centers worldwide.

Forty-three newborn screening centers from 38 countries took part in the survey. The results of our study showed that COVID-19 impacted the NBS programs, at least partially, in 29 out of 38 responding countries. The majority of the screening centers experienced a broad spectrum of difficulties and 65.7% were affected most prominently in the second wave of the pandemic. Delays and unreliability in the postal service as well as flight cancellations caused delays in samples arriving into screening centers and with the provision of laboratory equipment and reagents. The availability of laboratory staff was reduced due to infection, quarantine or reassignment within the healthcare facility. Sample collection at home, second-tier tests and follow-up were also affected. Social restrictions and the interruption of public transport added to these difficulties. Telemedicine emerged as important tool for maintaining outpatient care while limiting direct patient contact. The attention of the public health systems generally shifted from the provision of NBS services to COVID-19 related care. Only a limited number of centers managed to retain a fully functioning NBS program. Good practice examples were performed in Victoria, Australia and the UK.

The long-term effects of the pandemic on the well-being of children with disorders typically diagnosed by NBS remains to be reported. As the pandemic might be ongoing or could reoccur in future years, it might be useful to develop guidelines to protect these valuable services.

O05. **Advances in Mass Spectrometry-Based Newborn Screening: Quadrupling the Throughput of First-Tier Screening and Same Day, Same Instrument, Second-Tier Screening Using Intelligent Reflex**

Konstantinos Petritis, Samantha Isenberg, C. Austin Pickens and Carla Cuthbert

Centers for Disease Control and Prevention, Newborn Screening and Molecular Biology, Chamblee, GA, USA

Mass spectrometry revolutionized the field of newborn screening allowing high specificity and multiplexed assays to drive a significant expansion of disorders screened globally. Presently, dozens of disorders can be simultaneously screened from just one dried blood spot punch. More recently, the introduction of second-tier screening using LC-MS/MS further improved NBS by increasing specificity and sensitivity of the assay. However, despite recent advances in tandem mass spectrometry (MS/MS), liquid chromatography (LC), automation and data analytics, the general workflow and throughput have remained similar for decades. The second-tier screening rate of adoption by NBS laboratories has been relatively slow, with recent surveys reporting major considerations by NBS labs are the negative impact in timeliness, as well as the need for additional capital instrumentation and specialized personnel.

This study presents a novel workflow using state-of-the-art LC components such as a dual-needle/dual loop autosampler coupled to fast scanning triple quadrupole MS, which increases the throughput from around 2 min to 30 s per specimen. The autosampler used in our study can complete the needle wash and aspirate the next sample, while the previous sample is being analyzed, thus, eliminating analytical overhead experienced with single syringe/single loop autosamplers. Additionally, we present a software driven intelligent reflex option that identifies presumptive positives and reflexes these screen positive specimens from Flow Injection Analysis (FIA)-MS/MS to second-tier liquid chromatography (LC-MS/MS) analysis; using a multi-selector switching valve without any manual intervention. This instrumentation setup not only quadrupled the throughput of first-tier screening to roughly 120 samples per hour, but also allowed second-tier screening to subsequently be performed on the same platform, on the same day, assuming roughly four 96 well plates per day per instrument.

O06. **Implementation of a Combined Screening Test for SCID and SMA in the Netherlands Based on Quantitative PCR**

Els Voorhoeve, Pim Vergeer, Hennie Hodemaekers, Sandra Imholz, Paul van Ommeren, Marie-Louise Heijnen, Evelien Kemper, Henk Engel and Henny Lemmink

National Institute for Public Health and Environment, Biologicals, Screening & Innovation, Bilthoven, The Netherlands

Spinal muscular atrophy (SMA) is a rare and devastating autosomal recessive disease. Most SMA patients carry a pathogenic homozygous deletion in exon 7 of the SMN1 gene. New disease-modifying treatments recently became available and clinical studies show that early treatment is related to a better outcome. The Dutch Health Council advised the Dutch Secretary of State for Health to include SMA in the Dutch Newborn Screening program (NBS). This advice was based on the Wilson and Jungner criteria. The decision was made to follow the advice and the National Institute for Public Health and Environment estimated to be ready to screen for SMA not later than 1 October 2022.

As of 2021, a screening test for severe combined immunodeficiency (SCID) was implemented in the Dutch NBS. A kit developed by ImmunoIVD, based on real-time polymerase chain reaction (qPCR) and the measurement of the number of T-cell receptor excision circles (TREC), is used for the SCID screening. ImmunoIVD extended this kit with probes specific for exon 7 of the SMN1 gene to provide a newborn screening test for both SCID and SMA. The result of the SMN1 test is expressed as the number of PCR cycles the exon 7 amplicon needs to pass the detection threshold (Ct). The homozygous deletion of exon 7 in the SMN1 gene is detected by a Ct value above the threshold value or no Ct value is measured at all with the SMN1 specific probes. 

A successful proof-of-concept experiment was performed on 324 neonatal Dried Blood Samples (DBS), including samples of four SMA patients and including nine low TREC samples. Subsequently a validation/verification study is set up to prepare for the implementation of this combined test in the five Dutch screening laboratories. The relevant validation parameters for this assay, which combines quantitative aspects (number of TREC) and semi-quantitative aspects (Ct of SMN1 above or below the threshold value), will be discussed and the first results of the validation study will be presented.

O07. **Statewide Newborn Screening for Spinal Muscular Atrophy: The Wisconsin Experience**

Mei Baker, Sean Mochal, Sandra Dawe, Amy Wiberley-Bradford, Michael Cogley, Bethany Zeitler, Zachary Piro, Mathew Harmelink and Jennifer Kwon

Wisconsin State Laboratory of Hygiene, University of Wisconsin School of Medicine and Public Health, Madison, WI, USA

Spinal muscular atrophy was recently added to the Wisconsin newborn screening panel. Here we report our screening methods, algorithm, and outcomes. Methods: A multiplex real-time PCR assay was used to identify newborns with homozygous SMN1 exon 7 deletion, and those newborns’ specimens further underwent a droplet digital PCR assay for SMN2 copy number assessment. An independent dried blood spot specimen was collected and tested to confirm the initial screening results for SMN1 and SMN2. Results: From 15 October 2019 to 14 May 2021, a total of 93,834 newborns were screened for spinal muscular atrophy. Eight newborns screened positive for and were confirmed to have spinal muscular atrophy, making the Wisconsin spinal muscular atrophy birth prevalence 1 in 11,729. Of these six infants, three have two copies of SMN2, three have three copies of SMN2, and two have four copies of SMN2. Seven newborns received Zolgensma therapy, and one newborn received Spinraza therapy. Conclusions: Our screening method’s positive predictive value is 100%. This comprehensive approach, providing both timely SMN2 information and SMN1 and SMN2 confirmation as parts of the algorithm for spinal muscular atrophy newborn screening, facilitated timely clinical follow-up, family counseling, and treatment planning.

## 3. Poster Presentations

### 3.1. Endocrine Disorders

P01. **Analytical and Population Evaluation of the Automated Neonatal hTSH-FEIA Plus Kit from Labsystems Diagnostics**

Gustavo Borrajo

Fundación Bioquímica Argentina, Detección de Errores Congénitos, La Plata, Argentina

The availability of analytically and diagnostically reliable methods for the TSH measurement is a key requirement for an efficient neonatal detection of Congenital Hypothyroidism (CH). Automation feasibility, high throughput, and low costs are also very important features to consider when deciding on a certain method. Here, results of analytical and population evaluation of the automated Neonatal hTSH FEIA Plus kit using an NS2400 System from Labsystems Diagnostics Oy (LD) are presented. Recovery and precision were determined using control materials (CM) from the NSQAP–Centers for Disease Control and Prevention (CDC) at three levels (mean 14.1, 23.4 and 48.9 µU/mL), and the kit CM (LD-CM) at two levels (mean 8.9 and 35.6 µU/mL). Population evaluation was made analyzing 575 normal TSH specimens from non-CH newborns (NB) and 22 abnormal TSH specimens previously tested with the AutoDELFIA Neonatal hTSH kit from PerkinElmer (PE): 18 from non-CH and 4 from CH NB, TSH ranges [11.0–17.0] and [11.3–212.3] µU/mL respectively. LD and PE results were correlated and compared. Intra-assay CV’s were <12.9, <7.6 and <6.9% for the CDC-CM (3 runs, n = 20). Inter-assay CV’s were 8.9, 8.5 and 6.5% for the CDC-CM, and 15.6 and 7.9% for the LD-CM (10 runs, n = 2). Analytical recovery using CDC-CM was 119.5, 110.8 and 91.0%. The method was linear until around 80.0 µU/mL. Method correlation was appropriate (TSHLD = 0.802 TSHPE + 0.811, r = 0.810, n = 575), demonstrating a slight LD TSH underestimation regarding PE. The preliminary LD cutoff value was 7.4 µU/mL (99.0th percentile). 569/575 non-CH NB with normal PE TSH were detected as normal by LD (98.96%), 15/18 non-CH NB with abnormal PE TSH were detected as abnormal by LD (false positives), and 4/4 CH NB were detected as abnormal by LD (100%).

The evaluated Neonatal hTSH FEIA Plus kit showed an appropriate analytical and diagnostic performance, and the NS2400 autoanalyzer offered an acceptable level of automation, being apt to be introduced into the CH newborn screening routine.

P02. **Newborn Screening for Endocrine Disorders: Belgian Experience with ZenTech s.a. Devices**

Madeleine Boulanger, François Boemer, Roland Coenen, Géraldine Luis and Delphine Debois

ZenTech, R&D-Validation, Angleur, Belgium

Neonatal screening for congenital hypothyroidism (CH) and congenital adrenal hyperplasia (CAH) are two endocrine disorders commonly integrated in newborn screening programs worldwide. The screening is generally ensured by quantifying, respectively, TSH and 17-OHP in newborns’ dried blood spots.

In the Liège area (Belgium), newborn screening is centralized in the Biochemical Genetics Laboratory (CHU of Liège), which uses the NEONATAL TSH Screening ELISA and ELIZEN 17-OHP Screening Assay devices, both CE-marked and manufactured by ZenTech s.a.

The aim of the presentation is to show a feedback from a three-year-long use of these devices, on more than 15,000 samples for each parameter. These data were used to assess the clinical performance of the devices (diagnostic sensitivity, diagnostic specificity, etc.) according to the UE 2017/746 regulation (IVD-R, Annex I).

P03. **Neonatal Screening for Congenital Hypothyroidism: The Importance of a Third Sample in Large Prematures**

Enrique Melguizo Madrid, Ana Isabel Alvarez Rios, David Nuñez Jurado and Carmen Delgado Pecellín

H. U. Virgen del Rocío de Sevilla, Metabolic Diseases Unit, Sevilla, Spain

Introduction: Congenital hypothyroidism (CH), caused by insufficient thyroid hormone production, is the most common endocrine development disorder and the most common preventable cause of mental retardation. Among the current challenges of neonatal screening for CH is the screening of preterm, underweight and newborn large premature (LP) (less than 31 gestation weeks (GW) or 1500 g of birth weight). In these cases, the primary CH may be masked mainly due to the immaturity of the hypothalamic-pituitary-thyroid axis. 

Objectives: Identify newborns LP affected by CH with delayed elevation of TSH and evaluate our detection strategy.

Material and Methods: A retrospective analysis of 239,777 newborn samples was performed. The strategy used includes a second TSH determination at two weeks of age in premature infants (<37 GW) or weight <2500 g) and a third in LP (weight <1500 g or <31 GW) infants when reaching 2500 g of weight or at discharge. A TSH > 15 µIU/mL was considered positive screening and serum thyroid function was evaluated. If TSH was between 7.5–15 µIU/mL, a second sample was required to evaluate its evolution and TSH > 7.5 µIU/mL in the second or third sample were also considered positive. The confirmatory tests were: serum TSH and FT4.

Results: 239,777 newborn babies were analyzed and 2266 were LP (0.95%). 27 of them had a TSH > 7.5 µIU/mL in the second sample and finally, five congenital hypothyroidisms andtwo2 subclinical hypothyroidisms were diagnosed. In addition, six patients presented a [TSH] > 7.5 µIU/mL in the third sample: one of them was exitus, one false positive, two subclinical hypothyroidisms, and two patients had a serum TSH concentration of 132 and 122 µIU/mL with decreased FT4, diagnosing CH. This assumes a 1:385 incidence of LP with delayed elevation of TSH. 

Discussion: We conclude that is important to repeat the CH screening in LP, in addition to 15 days, with a third sample at hospital discharge to avoid losing cases with late TSH elevation.

P04. **Reference Value of Thyroid-Stimulating Hormone (TSH) for Normal Newborn Babies in Yogyakarta, Indonesia**

Tri Ratnaningsih, Windarwati Windarwati and Putri Raudina Alifah

Universitas Gadjah Mada, Clinical Pathology and Laboratory Medicine, Yogyakarta, Indonesia

Background: Congenital hypothyroidism (HK) is one of the causes of mental retardation in children, which can be prevented by screening every newborn aged 2–4 days. This screening looks at thyroid-stimulating hormone (TSH) levels from a blood sample taken from the heel of a newborn. TSH levels for HK from each different population may have different reference values. The cut-off value > 20 mU/mL is still used; it will be classified as presumptive HK.

Purpose: This study aims to find the reference value of TSH levels in normal newborns in the Special Region of Yogyakarta

Methods: This study is an observational (non-experimental) quantitative descriptive study. Data were obtained by retrospective observation using congenital hypothyroid screening data in the Special Region of Yogyakarta in 2017, obtained from the Department of Clinical Pathology, Faculty of Medicine, Public Health, and Nursing UGM. Total subjects were 4631 samples, with samples that met the research criteria was 1072 samples of newborns with healthy categories (normal weight and term).

Results: From this study, the reference value of TSH for normal newborns was 0.66–9.67 mU/L, with the upper limit being the cut-off value (9.67 mU/L). When compared with the cut-off value applied by the government (20 mU/L), more samples (n:45) required a confirmation test when using the cut-off value from this study. In addition, there was no significant difference in TSH levels in normal newborns with LBW and preterm newborns (*p* > 0.005).

Conclusion: The reference value of TSH for normal newborns is 0.66–9.67 mU/L. Decreasing the referral value will increase the likelihood that an infant with transient congenital hypothyroidism will receive further treatment.

Keywords: Reference Value, TSH, Congenital Hypothyroidism

P05. **Incidence of Congenital Hypothyroidism in the Republic of North Macedonia in Correlation with TSH Cutoff Level**

Violeta Anastasovska, Milica Pesevska, Mirjana Kocova, Elena Sukarova-Angleovska, Nermina Fakovic and Senada Karishik

Department for Neonatal Screening, Faculty of Medicine, Ss. Cyril and Methodius University in Skopje, University Clinic for Pediatrics, Skopje, North Macedonia

The incidence of congenital hypothyroidism (CH) worldwide ranging from 1:2000 to 1:4000 newborns. Lower cutoff levels in screening programs have led to an increase in the proportion of detected cases with transient hypothyroidism, leading to increase of the overall incidence of primary CH.

Data from a newborn thyroid screening program over a 19-year period (2002–2020) were analyzed. Total of 354,422 (97.2%) neonates were screened for thyroid-stimulating hormone (TSH) level in dried blood spot specimens taken 48 h after birth, using DELFIA method. A TSH value of 15 mU/L was used as the cutoff point until 2010 and 10 mU/L thereafter.

Primary congenital hypothyroidism was detected in 202 newborns with overall incidence of 1/1755, and female to male ratio 1.04:1. Among neonates with primary CH, 144 (71.29%) had permanent CH with female predominance (female to male ratio 1.4:1), and 58 had transient CH (28.71%) with male predominance (female to male ratio 0.49:1). The lowering of the TSH cutoff level almost doubled the incidence of primary CH (1/1480) compared to the incidence in the previous period (1/2489). It is interesting that the incidence of transient CH in the period with lower TSH cutoff level (1/3751) was twelve times higher than the CH incidence detected until 2010 (1/45,625). Opposite, the incidences of permanent CH, before (1/2632) and after (1/2365) changing the TSH cutoff level, were slightly different.

Our findings showed that the lower TSH cutoff values have impacted the increased incidence of primary CH, especially of the transient CH, in the country.

P06. **Coverage with Neonatal Thyroid Screening in the Republic of North Macedonia, during 2002–2020**

Milica Pesevska, Violeta Anastasovska, Mirjana Kocova, Elena Sukarova and Nermina Fakovic

University Clinic for Pediatrics, Department for Neonatal Screening, Skopje, North Macedonia

Neonatal thyroid screening is a reliable tool for early diagnosis and treatment of congenital hypothyroidism, the most common cause of preventable brain damage. A newborn thyroid screening program was established nationwide in 2007 in North Macedonia, after five years as a pilot study.

One screening center in the North Macedonia, located at the University Clinic for Pediatrics in Skopje, covers neonates born in 27 public and private birth centers all over the country. Thyroid-stimulating hormone (TSH) levels were analyzed from dry blood spots collected 48 h after birth on filter paper using the DELFIA method, during the period 2002–2020.

During this period, out of 364,574 live births 354,422 were screened. The coverage of the screened newborns was 97.2% on average, ranging from 91.1% in 2002 to 98.8% in 2020. There is continuously increasing rate of coverage in the last five years (98.3% in 2016, 98.2% in 2017, 98.3% in 2018, 98.6% in 2019 and 98.8% in 2020) as a result of improved education and training of the personnel in the births centers across the country. The biggest birth centers in the country, University Clinic of Gynecology and obstetrics (94.7%; 96.5%; 96.7%), and Clinical Hospital in Tetovo (96.8%; 98.7%; 98.8%), noticed increased coverage in the last three years.

TSH neonatal screening was satisfactorily implanted in our country with noticeably increased coverage in recent years. Diagnosis of congenital hypothyroidism in the first month of life ensure normal growth and development in children.

Key words: Coverage, Newborn screening, Thyroid-stimulating hormone

P07. Withdrawn by the authors

### 3.2. Cystic Fibrosis

P08. **Implementation of Cystic Fibrosis (CF) Newborn Screening (NBS) in Luxembourg: 3 Years’ Experience**

Anna-Maria Charatsi, Meriem Mastouri, Caroline Eisele, Flore Nzuangue, Patricia Borde, Marizela Kulisic, Dominique Bourgeois, Barbara Klink and Isabel de la Fuente Garcia

Centre Hospitalier de Luxembourg, Paediatrics, Pneumology, Luxembourg, Luxembourg

Introduction: CF NBS in Luxembourg was introduced in 1 January 2018. The implementation of the protocol was based on the characteristics of the Luxembourgish population, which is multi-ethnic, with 50% of habitants being non-autochthones.

Objectives: (1) To describe the CF NBS protocol in Luxembourg and evaluate its performance compared to recommendations of standards of care, (2) To calculate the incidence of CF in Luxembourg.

Methods: The Luxembourgish protocol for CF NBS relies on Immunoreactive Trypsinogen (IRT) on day 3 of life (D3) as the primary test. CFTR mutation analysis and a second IRT on D21 are performed in the case of high IRT (>60 ng/mL). CFTR mutation analysis is performed at the National Laboratory of Health with the CF-EU2v1 (Elucigene) panel which includes 50 pathogenic variants and four polymorphisms in CFTR gene. Patients screened positive are referred to the Pediatric National CF center where sweat test is performed for confirming or excluding the diagnosis of CF. The clinical follow up of patients takes place at the Pediatric National CF center which provides multi-disciplinary care. Genetic counseling is provided to all patients with pathologic genetic tests. 

Results: IRT was above the threshold level for 0.6% of live births (n = 45) in 2018; 0.97% (n = 70) in 2019; 1.38% (n = 105) in 2020. CF was diagnosed in nine infants (n = 3 in 2018; n = 4 in 2019; n = 2 in 2020). All patients had at least one CFTR mutation identified by the genetic panel. Identification of the second mutation by whole gene sequencing was required in four cases (4382delA, E664X, I502T, S489L). The overall calculated incidence of CF was 1/2454 live births. All infants with a confirmed CF diagnosis were seen by a CF specialist within 32 days of life (median: 28 days).

Conclusion: CF NBS was introduced in Luxembourg in 2018 and is in line with current recommendations for standards of CF care. The incidence of CF in infants born in Luxembourg is comparable to neighboring countries. Further assessment is required in order to validate the diagnostic protocol.

P09. **Neonatal Screening Program for Cystic Fibrosis in Western Andalusia: Experience after 10 years of Implementation**

Ana Isabel Alvarez-Rios, Enrique Melguizo Madrid, Isabel Delgado Pecellin, Maria Esther Quintana Gallego and Carmen Delgado Pecellin

H. U. Virgen Del Rocio, Metabolic Diseases Unit, Sevilla, Spain

Introduction: In 2011, Cystic Fibrosis (CF) was included in the Neonatal Screening Program (NSP) of our autonomous community. The objective of our work is to analyze the results obtained 10 years after its implantation and to calculate the prevalence in our population.

Method: The study was carried out in the Metabolic Diseases Unit of the Virgen del Rocío University Hospital. From May of 2011 to May of 2021 were analyzed 426,600 samples of newborns. The detection of CF was based on the determination of immunoreactive trypsin (IRT) using the delayed-time immunofluorescence technique (DELFIA^®^) (PerkinElmer^®^). The strategy used was IRT1/IRT2/sweat test (ST). The cut-off point for IRT1 was established at 61 ng/mL. If it was higher, a new sample was extracted 25 days after birth whose cut-off point or IRT2 was 40 ng/mL. If it was higher, the patient was referred to the Cystic Fibrosis Clinical Reference Unit where the ST was performed and if it was positive or doubtful, it was confirmed by a genetic study with the Elucigene^®^CF-EU2v1 kit of 50 mutations (Elucigene Diagnostics) and/or sequencing of the CFTR gene responsible for CF.

Results: We detected 412 positive screenings, 50 of them were diagnosed as affected by CF. Furthermore, during this period there were nine false negatives. The prevalence of this disease in the analyzed period is 1:7231.

Conclusions: CF screening in our Autonomous Community has made it possible to improve the prognosis and quality of life of people affected by this disease.

The neonatal screening program has allowed a large number of newborns to benefit from the early detection of certain serious congenital diseases and improve the morbimortality of those who suffer from them.

P10. **Evaluation of the Newborn Screening Program for Cystic Fibrosis in Catalonia with the Incorporation of the Pancreatitis-Associated Protein (PAP) as Second-Tier Test**

Rosa Maria Lopez-Galera, Ana Argudo-Ramirez, Jose Manuel Gonzalez de Aledo-Castillo, Abraham Jose Paredes-Fuentes, Sonia Pajares-Garcia, Giovanna Delgado-López, Jose Eduardo Flores-Jimenez, Esther Ramon-Moreno, Nieves Castillo-Martínez, Judit Perez-García, Celia Badenas-Orquin, Silvia Gatner-Tizzano, Maria Cols-Roig, Oscar Asensio-de la Cruz, Laia Asso-Ministral, Blanca Prats-Viedma, Judit Garcia-Villoria and Jose Luis Marin-Soria

Hospital Clínic of Barcelona, Biochemistry and Molecular Genetics Service, Barcelona, Spain

Introduction: Cystic fibrosis (CF) was implemented in Catalonian newborn screening program (NBS) in 1999, with a double sample strategy for immunoreactive trypsin (IRT1 + IRT2), with a specificity of 75% and positive predictive value (PPV) of 4%. In November 2017, the pancreatitis-associated protein (PAP) was incorporated as a second tier test.

Objectives: Evaluate the effectiveness of our NBS program for CF after the incorporation of PAP as a second-tier test.

Material and methods: From November 2017 to June 2021, IRT1 (samples at 48 h) and IRT2 (samples between 21–30 days) were analyzed. PAP was performed in positive IRT1 samples. Two strategies were applied, from November 2017 to March 2020, the strategy was (IRT1 + PAP + IRT2) followed by a DNA analysis and sweat test to all positive detections; from April 2020 to April 2021 the strategy was (IRT1 + PAP + DNA), only newborns with one or two mutations or [IRT1 ≥ 150 ng/mL] without mutations the sweat test was performed. Sensitivity, specificity, PPV and average age of newborn at diagnosis were calculated.

Results: 221,755 samples from newborns were analyzed. Four-hundred eighty-nine positive cases were detected, they were confirmed: 23 CF, 33 CFSPID (CF screening positive inconclusive diagnostic) and 69 carriers. The sensitivity was 100% with both strategies. The best results were obtained with the second strategy: the specificity, PPV and average age at diagnosis achieved were 99.6%, 7.6%, and 24 days, respectively.

Conclusions: The results showed that the inclusion of PAP as a second-tier test achieves a high specificity and sensibility, identifies a low number of CFSPID and healthy carriers, and reach a favorable average age at diagnosis. However, the PPV of the program still needs to be improved.

P11. **Introduction of Neonatal Screening for Cystic Fibrosis in the Republic of North Macedonia**

Violeta Anastasovska, Milica Pesevska, Stojka Fustik and Ana Stamatova

Department for Neonatal Screening, Faculty of Medicine, Ss. Cyril and Methodius University in Skopje, University Clinic for Pediatrics, Skopje, North Macedonia

The newborn screening for cystic fibrosis (CF) in North Macedonia was started as a pilot study in 2018, and become national program from April 2019. Because early testing and treatment of CF can lead to improved health, the screening test must be conducted as soon as possible.

Immunoreactive trypsinogen (IRT) was measured from dry blood spots collected 48–72 h after birth on filter paper using the DELFIA method (referent value 70 ng/mL). Second IRT test was preformed after 21 day of birth (referent value 45 ng/mL). After positive second IRT test a sweat test was proceed. Final diagnosis is done by molecular testing on CFTR gene.

In the period from 2018 to May 2021, a total of 48,315 newborns were screened and CF incidence of 1:2842 was obtained. During this period, 225 newborns were called for second IRT test (0.47%) from which 160 had normal IRT levels and sweat test was done on 65 newborns. False positive were 48 (0.1%), and cystic fibrosis was detected on 17 (0.04%) newborns. CF diagnosis in newborns with positive sweat test was confirmed by molecular CFTR analysis, and F508del mutation was found as most frequently (70.6%). Among the CF newborns 11 were males and six females. According to ethnicity, Albanians newborns (70.6%) compared to Macedonians (29.4%) were dominant probably as a result of consanguinity marriages.

These results put into sharp importance of newborn screening for CF. It is equally important to ensure that programs are established with careful consideration of the implications for the population.

P12. **Cystic Fibrosis: Full CFTR Sequencing for All Hypertrypsinemic Infants in Norway**

Emma Lundman, Asbjørg Stray-Pedersen, Janne Strand and Rolf D. Pettersen

Oslo University Hospital, Norwegian National Unit for Newborn Screening, Oslo, Norway

The Cystic fibrosis patient population of Norway has long been known to have a lower frequency p.Phe508del homozygotes compared to other parts of Europe. Thus, when implementing CF newborn screening in 2012, a survey of the most common patient variants indicated that it would be beneficial to use a large variant panel (Luminex CFTR 71 variants assay) with additional Sanger analysis of common Norwegian patient CFTR variants (Lundman, Gaup et al. 2016). As new technological advances allowed for a more extensive variant evaluation, a three tier NGS workflow was adopted from 2015. Second tier MiSeqDx CFTR 139 variants analysis was followed by another NGS method, full gene sequencing of the carriers using IonAmpliSeq CFTR panel. However, the costly and time-consuming workflow gave long lead times for infants carrying rare variants. (manuscript in preparation)

As of 2021, a two-tier workflow was implemented to improve timeliness and comprehensiveness in the screening program.

All infants are offered newborn screening after 48 h of age. The first-tier test screen for elevated IRT (Immunoreactive trypsinogen), using the GSP (Perkin-Elmer). The cut-off at 38 ng/mL captures approximately 5% of samples. As a second tier, DNA is extracted from the same dried blood spots and sequenced over the whole CFTR gene using MiSeqDx and TruSight Cystic Fibrosis Clinical Sequencing Assay as a second tier.

This new era with full variant visibility combined with high sample throughput is expected to yield valuable insights into the spectrum of CFTR variants that can be observed in a population.

The results of the first months of full gene screening will be presented with regards to response time, variant interpretations and insights.

Ref: Lundman, E., H. J. Gaup, E. Bakkeheim, E. J. Olafsdottir, T. Rootwelt, O. T. Storrosten, and R. D. Pettersen (2016). “Implementation of newborn screening for cystic fibrosis in Norway. Results from the first three years.” J Cyst Fibros 15(3): 318–324.

### 3.3. Lysosomal Storage Disorders

P13. **Newborn Screening for LSDs in Brazil: Pilot Study Update**

Francyne Kubaski, Ines Sousa, Tatiana Amorim, Juliana Badaró, Danilo Pereira, Ana Carolina Brusius-Facchin, Alice B. T. Netto, Joe Trometer, Alexandre Souza, Enzo Ranieri, Giulia Polo, Xinying Hong, Alberto Burlina, Michael Gelba and Roberto Giugliani

UFRGS/HCPA/INAGEMP, PPGBM/Medical Genetics Service, Porto Alegre, Brazil

Introduction: This report provides an update on the pilot newborn screening program for six lysosomal storage diseases (LSDs) in Brazil. 

Materials and methods: Dried blood spots (DBS) samples of 20,000 unselected newborns from the state of Bahia are being analyzed by the Neo-LSDä kit (Perkin-Elmer) by liquid chromatography/tandem mass spectrometry (LC-MS/MS) with a Xevo TQ-S micro (Waters). Activities of a-iduronidase (IDUA) glucocerebrosidase (ABG), sphingomyelinase (ASM), galactocerebrosidase (GALC), a-galactosidase (GLA), and a-glucosidase (GAA), were measured. The samples with low enzyme activity were submitted to the evaluation of specific biomarkers: glycosaminoglycans-GAGs, lyso-Gb1, lyso-SM, psychosine, or lyso-Gb3 (no marker for Pompe disease in DBS so far), again with LC-MS/MS, followed by molecular analysis of the relevant gene by next-generation sequencing (NGS). All these tests were performed in the same DBS sample. 

Results: So far, samples from 19,636 newborns have been analyzed. One sample had low IDUA activity, but analyses of GAG species were normal, and NGS of the IDUA gene revealed two variants associated with pseudodeficiency and one VUS. Two male newborns were confirmed with Fabry disease (low GLA, elevated Lyso-GB3, and pathogenic variants in the GLA gene). Four newborns were identified with Pompe disease (low GAA levels and pathogenic mutations in the GAA gene), and in the other three the molecular analysis is pending. 

Conclusions: In 19,636 newborns of Bahia state, Northeast Brazil, we have already identified four cases of Pompe (1/4909), and two cases with Fabry (1/9818), in addition to one with IDUA pseudodeficiency. This study indicates that using enzyme assay, biomarker measurement, and genetic studies, screening, and diagnosis is possible with a single DBS sample, simplifying and speeding up the overall process.

P14. **Evaluation of Multiple Methods of Glycosaminoglycan Analysis in Newborn Dried Blood Spots to Support Newborn Screening for Mucopolysaccharidoses**

Michael Gelb and Zackary Herbst

University of Washington, Chemistry, Seattle, WA, USA

Newborn screening for MPS-I and -II have started in several newborn screening labs worldwide. All labs measure the activity of the relevant lysosomal enzyme in dried blood spots (DBS) as the first step of newborn screening. Because of pseudodeficiencies, false positives are prevalent. In the case of MPS-I, analysis of glycosaminoglycan (GAG) fragments in dried blood spots as a second stage test and as part of newborn screening reduces the false positive rate to near zero.

We have been analyzing GAG fragments in newborn DBS from patients diagnosed with all types of MPS. We have been testing, in parallel, two different methods for GAG analysis. One method is the classical one in which full length GAGs are digested with bacterial enzymes, and the internal disaccharides are analyzed by LC-MS/MS. The second method is to quantify by LC-MS/MS the non-reducing end GAG fragments generated in human tissues and found endogenously in DBS. For MPS-I, the non-reducing end method is superior to the classical method as the separation of biomarker levels for patients versus the reference range is much larger. The GAG data are more conclusive than genotyping analysis for predicting disease status and for reducing false positives. Available data for the other MPS disorders is being collected now and will be available in time for presentation at ISNS 2021 Conference.

P15. **Significant Unmet Need in Infants with Mucopolysaccharidosis VII and Non-Immune Hydrops Fetalis: A Summary of Cases**

Deborah Marsden, Camille L. Bedrosian, Tobin Chettiath and Kirin Jamison

Ultragenyx Pharmaceutical Inc., Medical Affairs, Novato, CA, USA

Non-immune hydrops fetalis (NIHF) occurs in ~40% of infants born with the ultra-rare lysosomal storage disease mucopolysaccharidosis VII (MPS VII). NIHF is often fatal among infants with MPS VII, with a one-year mortality rate of >40%. In some cases, a prenatal diagnostic evaluation may not include lysosomal storage diseases.

We compiled real-world data on 37 treatment-naïve patients: 25 (68%; 25/37) with confirmed NIHF, 8 (22%; 8/37) without NIHF, and 4 (11%; 4/37) with unknown NIHF status. Requests were received for vestronidase alfa as enzyme replacement therapy prospectively for in utero diagnoses or for infants diagnosed at <1 year old.

Of the 25 patients with NIHF, 13 (52%; 13/25) died before treatment (including three patients who died in utero) and two (8%; 2/25) died following complications of MPS VII during treatment with vestronidase alfa (approximates a 60% one-year mortality rate). Ten (40%; 10/25) patients with NIHF survived >1 year, including six who received vestronidase alfa before age 1 and four who received vestronidase alfa after age 1. All eight patients without NIHF survived >1 year (0% one-year mortality rate), seven of whom received vestronidase alfa. Three of the four patients with unknown NIHF status did not survive >1 year, and the outcome of the fourth patient is unknown. None of the patients with unknown NIHF status received vestronidase alfa. Overall, 19 patients received vestronidase alfa, including 12 with NIHF and 7 without NIHF. No patients received in utero treatment.

These results demonstrate an urgent unmet need in patients with MPS VII who have NIHF and underscore the importance of newborn screening and MPS VII confirmation to allow for timely consideration of treatment options, including vestronidase alfa. Additional strategies, such as in utero administration of vestronidase alfa, following prenatal diagnosis, or increased dosing of vestronidase alfa for infants diagnosed <1 year of age require evaluation, and may address the unmet need.

### 3.4. Haemoglobinopathies

P16. **Neonatal Screening Experience Sickle Cell Anemia and Other Hemoglobinopathies in Western Andalusia**

David Nuñez Jurado, Ana Isabel Alvarez Rios, Salvador Payan Pernia, Margarita María Jiménez Jambrina, Enrique Melguizo Madrid and Carmen Delgado Pecellín

H. U. Virgen del Rocío de Sevilla, Metabolic Diseases Unit, Sevilla, Spain

Background: Sickle cell anemia (FA) and other hemoglobinopathies were incorporated into the Andalusian screening panel in November 2018. FA is a hereditary disorder caused by the presence of hemoglobin S (HbS), the result of a specific mutation that it affects codon 6 of the betaglobin chain. Under hypoxic conditions, polymerization of HbS occurs, leading to vasocclusive crises and hemolytic anemia. Affected children are very susceptible to severe bacterial infections and present a high degree of morbimortality due to splenic sequestration and bacterial septicemia. 

Material and Methods: A descriptive and retrospective study carried out at our hospital between January 2019 and December 2020 for the detection of hemoglobinopathies in newborn screening. All specimens were first examined by HPLC. Profiles consistent with FA, sickle cell trait, β-thalassemia, or variant hemoglobins other than HbS were subsequently analyzed by capillary electrophoresis (CE) as a confirmatory method. 

Results: During our study period, 90,872 live newborns were screened for FA. Among these, we diagnosis FA as homozygous HbSS in six cases, heterozygous HbSC in 2 and HbSD in 1. Additionally, we identified heterozygous for HbS in 180 cases, HbC in 57, HbD and E in four and other variants such as HbG in six cases and HbO-Arab and HbC/β-thalassemia in two. A complete correlation between HPLC and CE was obtained in the diagnosis of FA and detection of HbS and C. However, major discrepancies among these techniques were noted to detect HbD, E, G, and O-Arab. 

Conclusions: An adequate FA screening program require at least two different methods combined in order to identify the hemoglobin present with sufficient certainty. Moreover, even though software solutions for HPLC suggest a pattern, it has to be verified, confirmed with another technique such as CE, and evaluated in the context of the study population for a correct interpretation of the chromatograms.

P17. **Incidence of Sickle Cell Disease and Major Thalassemic Syndromes in the Newborn Screening Program of Catalonia in the Period 2015–2020**

José Manuel de Aledo, Sonia Pajares García, Rosa María López Galera, Ana Argudo Ramírez, David Beneitez Pastor, Cristina Martínez Carreira, Yania Quintero, Tatiana Collado, Ariadna Muniente Caralt, Adoración Blanco Álvarez, Ana Ortuño Cabrero, Bárbara Tazón Vega, Thais Murciano Carrillo, Laura Murillo Sanjuan, Pablo Velasco Puyo, Cristina Díaz de Heredia, Cristina Gutiérrez Valle, Mar Mañú Pereira, Rosa María Fernández Bardón, Laia Asso Ministral, Blanca Prats Viedma, Carmen Cabezas, Antonia Ribes Rubió, Judit García Villoria and José Luis Marín Soria

Hospital Clínic de Barcelona, Biochemistry and Molecular Genetics, Barcelona, Spain

Introduction: SCD was included in the Spanish National Health System’s common portfolio for Newborn Screening Programs (NSP) from 2013. In addition to SCD, European consensus guidelines recommend reporting B-thalassemia (B-tal) in NSPs. Newborn screening for SCD began in 2015 in Catalonia. The aim of this work is to present the incidence, genotypes, and phenotypes of newborns affected by SCD and major thalassemic syndromes in Catalonia between 2015 and 2020, as well as the origin of the mothers of newborns affected by SCD and the incidence of HbS, HbC, HbD, and HbE carriers among newborns in our population.

Methods Between 2015 and 2020, dried blood spot samples of all newborns in Catalonia were analyzed by capillary electrophoresis at the Hospital Clínic de Barcelona. Confirmatory diagnosis were carried out by HPLC analysis and additional molecular studies at Vall d’Hebron Hospital, Barcelona.

Results The total number of newborns screened was 392,858 and the total diagnosis of SCD was 123. The overall incidence of SCD was 1/3193 newborns. 76.4% of cases presented the FS phenotype (67.5% BS/BS and 8.9% BS/B0 or BS/B+). The remaining 23.6% corresponded to the FSC phenotype (BS/BC). The origin of the mothers of newborns affected by SCD was 75.6% from Sub-Saharan Africa, 8.1% from North Africa, 10.6% from Central America, 4.9% from South America and 0.8% from Spain. The incidence of B-tal was 1/78,572 newborns and a-thalassemia 1/130,953 newborns. The incidence of hemoglobin C homozygosity was 1/32,738 newborns, hemoglobin D 1/130,953 newborns and hemoglobin E 1/392,858 newborns. The overall incidence of carriers was 1/105 newborns, being Hb S the most common (1/141).

Conclusions The migratory movements over the previous decades have led to SCD becoming the second most detected disease in our NSP. There has also been a corresponding and significant increase in Hb S carriers. The introduction of SCD in NSPs has allowed early detection, the application of prophylactic measures, and the reduction of morbidity and mortality.

P18. **Two Years of Newborn Screening for Sickle Cell Disease in Alberta, Canada**

Janet Zhou, Ross Ridsdale, Lauren MacNeil, Margaret Lilley, Stephanie Hoang, Susan Christian, Pamela Blumenschein, Vanessa Wolan and Iveta Sosova

Alberta Precision Laboratories, Genetics and Genomics, Edmonton, AB, Canada

Background: Sickle cell disease (SCD), a group of inherited red blood cell (RBC) disorders caused by a pathogenic variant in the beta-globin gene, can lead to lifelong disabilities, early mortality, or both. The morbidity and mortality associated with SCD can be reduced by early preventive measures. The NBS laboratory in Alberta (AB) began screening for SCD in April 2019. The primary conditions screened for are sickle cell anemia (HbSS), HbS/beta-thalassemia and HbSC disease. In addition, the decision was made to implement a universal disclosure policy for sickle cell trait (SCT) and include SCT results in the final NBS result. The objective of this study was to retrospectively evaluate the first two years of performance of NBS for SCD in AB.

Methods: Hemoglobins, eluted from a single 3.2 mm disk, were analyzed by cation exchange high performance liquid chromatography using the Bio-Rad™ VARIANT nbs analyzer. All specimens from infants who received a RBC transfusion (RBCT) prior to the first NBS specimen collection were analyzed concurrently by targeted sequencing of the HBB gene.

Results: Between 1 April 2019 and 31 March 2021, 99,606 of 100,191 infants registered in AB were screened. Of those screened, 241 infants received a RBCT prior to the initial NBS sample collection, and two received intrauterine fetal transfusions. In total, 60 infants had a positive screen for SCD. Diagnostic follow up testing confirmed SCD in 45 infants suggesting a local SCD incidence of 1:2200 births. Additionally, six infants had a false positive screen including three who were HbS carriers, two who carry a variant in alpha gene, and one who carries an unknown beta chain variant. The outcome for nine infants is still pending. We are unaware of any false negative screens. Finally, 749 infants were identified to have SCT, one of them was a transfused infant, resulting in a carrier frequency of 1 in 133 in AB.

Conclusions: Our screening algorithm for SCD enables detection of affected newborns within days after birth, independently of RBCT status.

### 3.5. Duchenne Muscular Dystrophy

P19. **Assessment of International Newborn Screening Programs for Duchenne Muscular Dystrophy**

Katherine L. Beaverson, Helene Schluep and Safiyya Gassman

Pfizer Inc., Rare Disease Research Unit, Cambridge, MA, USA

Introduction and Objectives: Newborn screening (NBS) may help to identify individuals at risk of Duchenne muscular dystrophy (DMD) several years earlier than a typical diagnosis; however, there are currently no formal programs for DMD NBS. The objective of this analysis was to evaluate the international NBS landscape for DMD to increase the understanding of previous NBS efforts, share learnings from pilot programs, and identify opportunities to advance NBS for earlier diagnosis and care management of DMD.

Methods: This was a survey and analysis of existing NBS policies and programs conducted in 10 countries: Brazil, Canada, China, France, Germany, Italy, Japan, Spain, the United Kingdom, and the United States. Assessments included: existence of regional and/or provincial NBS programs; which diseases were currently screened and the process for inclusion of a disease; pilot programs for DMD started and/or completed; reasons for discontinuation of programs; and challenges for inclusion of DMD. Countries were assessed based on the status of their NBS programs for DMD with findings further informed by country stakeholder insights on legislation and policy. 

Results: DMD is not currently included in the NBS policy framework for any of the assessed countries. The analysis found that key criteria for DMD NBS programs include funding, accuracy/specificity of testing methodology, treatment availability, and supporting infrastructure for follow-up referrals and care of patients and caregivers. Past NBS pilot programs in some countries (Canada, Germany, United Kingdom) were terminated due to a lack of these perquisites. A resurgence of DMD NBS pilot programs following the emergence of new technology was observed in China, Italy, Spain, and the United States.

Conclusion: This analysis provides an opportunity to advance discussions around NBS for DMD and supports recommendations for future NBS pilot programs with a goal of improving diagnosis, care management, and quality of life for individuals and families with DMD.

P20. **Multi-Laboratory Evaluation of Dried Blood Spot Materials for Creatine Kinase MM Isoform Immunoassays to Detect Duchenne Muscular Dystrophy in Newborns**

Paul Dantonio, Norma Tavakoli, Brooke Migliore, Elisabeth McCown, Timothy Lim, Sunju Park, Michele Caggana, Katerina Kucera, Robert F. Vogt and Konstantinos Petritis

Centers for Disease Control and Prevention, Newborn Screening and Molecular Biology Branch, Division of Laboratory Scie, Atlanta, GA 30341, USA

Assays to detect elevated levels of creatine kinase MM isoform (CK-MM) in newborn dried blood spots (DBS) can be used to screen for Duchenne Muscular Dystrophy (DMD). Newborn screening (NBS) pilot studies for DMD are currently being conducted by the New York State NBS Program (NYS) and by the Early Check Program at Research Triangle Institute (RTI) International. Both studies use a time-resolved fluoroimmunoassay to measure CK-MM concentrations in newborn DBS specimens. The Newborn Screening Quality Assurance Program (NSQAP) at the US Centers for Disease Control and Prevention (CDC) produced a set of seven prototype DBS reference materials spiked with varying levels of CK-MM to encompass the expected normal and elevated ranges in typical and DMD-affected newborns. These prototype DBS were evaluated at weekly intervals over a 3-week period by CDC, NYS, and RTI; all three used the fluoroimmunoassay. CK-MM results for each level from the three laboratories showed similar means and variance values and the seven levels collectively included the expected ranges in typical and DMD-affected newborns. This NSQAP preparative method for CK-MM DBS appears to be suitable for production of external quality control materials.

P21. **Newborn Screening for Duchenne Muscular Dystrophy in Guangzhou, A Pilot Study to Facilitate Implementation**

Xiang Jiang, Yong-lan Huang, Fang Tang, Rui-dan Zheng, Xue-fang Jia and Cheng-fang Tang

Guangzhou Women and Children’s Medical Center, Guangzhou Newborn Screening Center, Guangzhou, China, Peoples Republic

Objective: To estimate the overall situation of Duchenne muscular dystrophy (DMD) screening in newborns in Guangzhou, China and the feasibility of using creatine kinase-MM (CK-MM) measurement as the screening biomarker for DMD. To describe the distribution of CK-MM between male and female newborns, and the effect of gestational age (GA), birth weight (BW) and age at sampling on the concentration of CK-MM.

Method: A total of 39,746 newborns including 21,506 males and 18,240 females were screened for DMD from June to September 2019 by measuring the CK-MM concentrations in dried blood spots (DBS) using the time-resolved immunofluorescence. The results were grouped in terms of GA, BW and age at sampling. Newborns with positive result (CK-MM ≥ cut-off value, 680 ng/mL) were recalled and a second DBS sample was collected from the infant to remeasure the CK-MM concentration. The patients with a positive result in the retest were analyzed for serum CK-MM and genetic mutations from whole blood.

Results: Two males were found to have exon duplications and were diagnosed with DMD. The incidence of males was 1/10,753. There was no significant difference of CK-MM concentration between male and female newborns. However, significant differences in CK-MM concentrations were observed among newborns in terms of GA, BW and age at sampling. Further linear regression analysis showed that CK-MM concentration was much more closely correlated with GA and age at sampling, with a minimal effect related to BW.

Conclusions: A Duchenne newborn screening approach was established in Guangzhou, China. The 3-step path for newborn DMD screening fits our current health care infrastructure. CK-MM distribution in males and females has a similar pattern, CK-MM concentration is affected by GA, BW, and age at sampling, with GA and age at sampling showing a stronger effect on CK-MM concentration in newborns than the BW. The efficiency of DMD screening might be improved by adjusting a multitier cut-off value according to GA and age at sampling.

### 3.6. MS/MS Detectable Disorders

P22. **Very Long-Chain Acyl-CoA Dehydrogenase Deficiency: High Incidence of Detected Patients with Expanded Newborn Screening Program**

Žiga Iztok Remec, Urh Grošelj, Ana Drole Torkar, Mojca Žerjav Tanšek, Vanja Čuk, Daša Perko, Blanka Ulaga, Tadej Battelino and Barbka Repič Lampret

University Medical Centre Ljubljana, University Children’s Hospital, Clinical Institute of Special Laboratory Di, Ljubljana, Slovenia

Very long-chain acyl-CoA dehydrogenase deficiency (VLCADD) is a rare autosomal recessive disorder of fatty acid metabolism with a variable presentation. The aim of this study was to describe five patients with VLCADD diagnosed through the pilot study and Slovenian expanded newborn screening (NBS) program that begun in 2018. Four patients were diagnosed through the NBS program with tandem mass spectrometry; one patient was previously diagnosed in a pilot study before the NBS implementation. Confirmatory testing consisted of acylcarnitines analysis in dried blood spots, urine organic acids profiling, genetic analysis of ACADVL gene, and enzyme activity determination in lymphocytes or fibroblasts. Four newborns with specific elevation of acylcarnitines and disease-specific acylcarnitines ratios diagnostic for VLCADD (C14:1, C14, C14:2, C14:1/C2, C14:1/C16) were confirmed with genetic testing: all were compound heterozygotes, two of them had one previously unreported ACADVL gene variant each (NM_000018.3) c.1538C>G; (NP_000009) p.(Ala513Gly) and c.661A>G; p.(Ser221Gly). In addition, patient diagnosed in the pilot study also had a specific elevation of acylcarnitines. Subsequent ACADVL genetic analysis confirmed compound heterozygosity. Enzyme activity was reduced in all five patients tested. In seven other newborns with positive screening results, only single allele variants were found in the ACADVL gene, so the diagnosis was not confirmed. Among these, two variants were novel, c.416T>C and c.1046C>A, respectively (p.Leu139Pro and p.Ala349Glu). In the first two years of the expanded NBS program in Slovenia, altogether 30,000 newborns were screened and four cases of VLCADD were diagnosed. The estimated VLCADD incidence was 1:7500 which was higher than that of the medium-chain acyl-CoA dehydrogenase deficiency cases in the same period. 

Our study provided one of the first descriptions of ACADVL variants in Central-Southeastern Europe and also reported on four novel variants.

P23. **Acylcarnitines under FIA-MS/MS Conditions**

C. Austin Pickens, Samantha Isenberg, Carla Cuthbert and Konstantinos Petritis

US Centers for Disease Control and Prevention, Newborn Screening and Molecular Biology Branch, Atlanta, GA, USA

Introduction: Homocystinuria results from enzymatic or B-vitamin deficiencies involved in catabolism of methionine to cystathionine, which elevates levels of total homocysteine (tHcy). Hcy contains a thiol group and reacts with other thiol containing groups to form disulfide bonds with proteins and small molecules, so disulfide reduction is needed to assess tHcy concentrations. Methionine is used as a first-tier marker for homocystinuria, but methionine concentrations physiologically vary in newborn blood and may impact false positive and false negative rates. 

Methods: Samples analyzed in this study were QC materials created using adult blood. Multiplexing tHcy into first- and second-tier assays was assessed using both derivatized and non-derivatized sample preparation. Various reducing and thiol-specific derivatizing agents were investigated for feasibility and compatibility with current workflows. Sample analysis methods include FIA-MS/MS and LC-MS/MS coupled to triple quad platforms (TQ), and FIA-MS/MS and capillary electrophoresis coupled to high resolution mass spectrometry (HRMS).

Results: Preliminary data using FIA-MS/MS indicated the presence of nominal isobaric parent and product ions, sharing the same quantitative transition as tHcy that cannot be resolved on TQ platforms. We achieved similar tHcy concentrations when comparing FIA-MS/MS HRMS acquired data to data acquired by second-tier LC-MS/MS coupled to TQ platforms. Thiol derivatizing agents were investigated to selectively derivatize Hcy’s thiol group, which removed interfering Hcy isobars and increased thiol-derivatized tHcy signal. Preliminary data indicated reducing agents and thiol derivatizing agents increase ion suppression under FIA conditions, which impacted quantification of compounds using surrogate internal standards.

Conclusions: The present study demonstrates progress toward multiplexing tHcy into current first-tier FIA-TQ-MS/MS platforms, along with challenges and possible alternative approaches.

P24. **A Rapid Combined Second-Tier Test for Isovaleric Aciduria and Glutaric Aciduria Type I**

Karin Engström, Henrik Åhlman and Rolf Zetterström

Karolinska University Hospital, Centre for Inherited Metabolic Diseases, Stockholm, Sweden

Isovaleric Aciduria and Glutaric Aciduria type I were, together with 19 other disorders, included in the Swedish Blood Spot Newborn Screening program in 2010. Since this extension we have used a commercial non derivatized kit for the flow injection analysis tandem mass spectrometry (FIA-MS/MS) for the detection of relevant amino acids and acylcarnitines.

False positive results in FIA-MS/MS occur due to the presence of isobaric compounds. A second-tier test in which a chromatographic separation is introduced prior to the mass spectrometric detection can be used to overcome this problem. This strategy has previously been described for isovaleryl (C5)-carnitine and its isobaric compound pivaloylcarnitine. Screening of Glutaric Acidemia type I is performed by measurement of elevated glutaryl (C5DC)-carnitine. In a non-derivatized assay, the isobaric compound 3-hydroxyhexanoyl (C6OH)-carnitine is one of the compounds that can cause false positive results.

We developed a simple second-tier test to separate the isobaric compounds of both (C5)-carnitine and (C5DC)-carnitine in the same run. The same extracted sample that were analyzed in the first-tier FIA-MS/MS analysis is diluted or re-suspended and then injected and in a 6-min run in which the isobaric compounds are chromatographically separated. No new punching or extraction is needed, and therefore a delay of the result in the case of a positive outcome is avoided. The analysis is qualitative, and retention time comparison with isotopically labeled analogues as well as with QC-samples are used for identification.

The second tier analysis separating isobaric (C5)-carnitines were crucial in Sweden since pivmecillinam is the first drug of choice for pregnant women with urinary infections. With a PPV value of 29% in our first million screened babies for GA1 an improvement is warranted.

P25. **Newborn Screening and Disease Variants Predict Neurological Outcome in Isovaleric Aciduria**

Lucy Henze, Ulrike Mütze, Florian Gleich, Martin Lindner, Sarah C. Grünert, Ute Spiekerkoetter, René Santer, Holger Blessing, Eva Thimm, Regina Ensenauer, Johannes Weigel, Skadi Beblo, Maria Arélin, Julia B. Hennermann, Thorsten Marquardt, Iris Marquardt, Peter Freisinger, Johannes Krämer, Andrea Dieckmann, Nathalie Weinhold, Mareike Keller, Magdalena Walter, Katharina A. Schiergens, Esther M. Maier, Georg F. Hoffmann, Sven F. Garbade and Stefan Kölker

University Hospital Heidelberg, Division of Child Neurology and Metabolic Medicine, Center for Child and Adolesc, Heidelberg, Germany

Objective: Isovaleric aciduria (IVA), an organic aciduria with severe (classic IVA) or attenuated phenotype (mild IVA), is included in newborn screening (NBS) programs worldwide. The long-term clinical benefit of screened individuals, however, is still rarely investigated.

Methods: National, prospective, observational, multi-center study of individuals with confirmed IVA identified by NBS between 1998 and 2018. Comparison to previously published cohorts of symptomatically diagnosed patients.

Results: Long-term clinical outcomes of 94 individuals with IVA were evaluated, representing 73.4% (for classic IVA: 92.3%) of the German NBS cohort. In classic IVA (N = 24), NBS prevented untimely death except in one individual with lethal neonatal sepsis (3.8%) showing the benefit of NBS compared to pre-screening-era (18.7–43.2%, P = 0.08, P = 0.0078, respectively). However, NBS did not completely prevent single (N = 10) or recurrent (N = 7) metabolic decompensations, 13 of them occurring already neonatally. IQ (mean ± SD, 90.7 ± 10.1) was mostly normal but below the reference population (P = 0.0022) and was even lower in individuals with severe neonatal decompensations (IQ 78.8 ± 7.1) compared to those without crises (IQ 94.7 ± 7.5; P = 0.01). Similar results were obtained for school placement. In contrast, individuals with mild IVA had excellent neurocognitive outcomes (IQ 105.5 ± 15.8; normal school placement) and a benign disease course (no metabolic decompensation, normal hospitalization rate), which did not appear to be impacted by metabolic maintenance therapy.

Conclusion: NBS reduces mortality in classic IVA, but does not reliably protect against severe neonatal metabolic decompensations, crucial for favorable neurocognitive outcome. In contrast, individuals with mild IVA had excellent clinical outcomes regardless of metabolic maintenance therapy, questioning their benefit from NBS. Harmonized stratified therapeutic concepts are urgently needed.

P26. **Establishment and Validation of Reference Values for Amino Acids and Acylcarnitines in Dried Blood Spots for Omani Newborns Using Tandem Mass Spectrometry**

Sulaiman Al-Riyami, Matar Al-Manei, Assad Al-Fahdi and Khalid Al-Thihli

Sultan Qaboos University Hospital, Biochemistry, Muscat, Oman

Objectives: The objective of this study was to establish a reference range for acylcarnitines (ACs) and amino acids (AAs) concentrations in dried blood spot (DBS) samples of Omani neonates and to evaluate the effect of age and gender on ACs and AAs. Methods: An electrospray-ionization tandem mass spectrometry (+ESI-MS/MS) was used to determine ACs and AAs concentrations in 1302 healthy newborns (0–7 days) delivered at Sultan Qaboos University Hospital (SQUH) between August 2008 and May 2009. Results: More than 50 biomarkers that allow diagnosis of various inborn errors of metabolism (IEMs) were measured and the 1st and 99th percentile values were determined and compared with published international data. In comparison with the 1st and 99th percentile reported by Collaborative Laboratory Integrated Report (CLIR), our results were comparable despite a much smaller sample size. We found that age had a significant effect on most ACs and AAs except decadienoylcarnitine (C10:2), decenoylcarnitine (C10:1), adipylcarnitine (C6-DC), palmitoylcarnitine (C16:1), steatoylcarnitine (C18), tyrosine (Tyr), phenylalanine (Phe) and valine (Val). Whereas, gender of the neonate had insignificant effect on most of ACs and AAs except C0, acetylcarnitine (C2), hexanoylcarnitine (C6), octanoylcarnitine (C8), malonylcarnitine (C3-DC), C10, dodecenoylcarnitine (C12:1), dodecanoylcarnitine (C12) and tetradecanoylcarnitine (C14:1). Conclusion: MS/MS is a highly effective tool for high throughput screening of IEMs. This study is the first to publish reference intervals for ACs and AAs from DBS samples of Omani newborns. The results may prove to be of significance when determining cut-off values that maybe considered for newborn screening in the near future.

P27. **Newborn Screening for MCAD Deficiency: Experience of the First Ten Years in Eastern Andalusia, Spain**

Raquel Yahyaoui, Adela Pozo, María José Aguilar, Javier Blasco-Alonso, Carmen Benito, Pedro Ruiz-Sala and Belén Pérez

Málaga Regional University Hospital, Laboratory of Metabolic Disorders and Newborn Screening, Málaga, Spain

Background Medium-Chain Acyl-CoA Dehydrogenase Deficiency (MCADD) is an autosomal recessive fatty acid oxidation disorder with a potentially fatal outcome. It is caused by mutations in the ACADM gene; the most prevalent mutation is c.985A>G. The objective of this study was to evaluate the prevalence, clinical course, and biochemical and molecular phenotype of MCADD cases detected in the first 10 years of newborn screening in our center.

Methods: From April 2010 to December 2020, the acylcarnitine profile, including C6, C8, C10, and C10:1, of 433,614 newborn DBS cards was measured by tandem mass spectrometry. Newborns with screen positive results were referred to physicians for further confirmatory testing (plasma acylcarnitine analysis and identification of biallelic pathogenic variants in the ACADM gene) and follow-up care.

Results: Twenty-six newborns were referred for confirmatory testing for with C8 values above the screening cutoff of 0.17 µmol/L. They had a mean level of 5.72 µmol/L (range 0.90–32.38). All 26 had elevated C6 levels and 18 also had elevated C10 values. 20 of the screen positive infants had an MCADD diagnosis confirmed by plasma acylcarnitine analysis. Molecular testing was available for 21 confirmed cases: 13 were homozygous for the common c.985A>G mutation, four were compound heterozygous for c.985A>G, and five had other mutations. The variant c.199T>C was not found in any patient. The average follow-up period was 3.5 years. Two patients were lost to follow-up during the first year. Two patients had a metabolic crisis; both were homozygous for the c.985A>G mutation. Six patients had at least one episode of hypoglycemia during follow-up. Ten patients required L-carnitine oral supplementation. The estimated prevalence of MCADD is 1:16,677 live births. 

Discussion: MCADD frequency in our center is comparable to reports from other newborn screening programs. Early detection and treatment have successfully prevented adverse health outcomes in our MCADD patients.

P28. **Seven Years Experience with Selective Newborn Screening for Inborn Errors of Metabolism in North Macedonia**

Violeta Anastasovska, Mirjana Kocova, Elena Sukarova-Angleovska, Milica Pesevska and Nikolina Zdraveska

Department for Neonatal Screening, Faculty of Medicine, Ss. Cyril and Methodius University in Skopje, University Clinic for Pediatrics, Skopje, North Macedonia

The development of tandem mass spectrometry screening permits increasing the capacity of potentially detectable congenital metabolic diseases by semi-quantitative detection of over 70 metabolites and their characteristic patterns. Although inherited metabolic diseases (IEM) constitute more than 100 different rare conditions, their cumulative incidence reach of approximately 1:1600–2000 newborns.

Neonatal screening for IEM was performed by measuring of 12 amino acids and 13 acylcarnitines (Chromsystems Diagnostics, Germany), in dried blood spot collected 48–72 h after births, using LC/MS/MS method, during the 2014–May 2021. 

Total of 36,076 newborns (23.6% of neonatal population for the study period) have been screened for IEM and incidence of 1/2004 have been obtained. In selective metabolic screening in North Macedonia are included neonates born in 12 out of total 27 birth centers all over the country which completely accomplished sample collection criteria. The newborns with a prolonged stay in intensive care units and indication for IEM were also included in the metabolic screening program. We detected a total of 18 newborns with IEM, of which eight had medium-chain acyl-CoA dehydrogenase deficiency (MCAD), 6 had phenylketonuria (PKU), and single newborn with maple syrup urine disease (MSUD), hypermethioninemia (MET), tyrosinemia type I (TYR I) and carnitine palmitoyltransferase I (CPT I) deficiency, respectively. Additionally, PKU was detected in three more children born in private birth centers which were screened at age 10 months, 18 months, and 2 years at the request of the Department of Neurology at the University Clinic for Pediatrics.

Neonatal metabolic screening is an important diagnostic tool for the diagnosis of various types of IEM, and it can provide substantial benefits to patients and their families. Early diagnosis is important not only for treatment but also for genetic counseling. Activities to cover all newborns in Macedonia are underway.

P29. **Expanded Newborn Screening Program in Slovenia Using Tandem Mass Spectrometry and Confirmatory Next-Generation Sequencing Genetic Testing**

Barbka Repic Lampret, Ziga Iztok Remec, Ana Drole Torkar, Mojca Zerjav Tansek, Vanja Cuk, Dasa Perko, Blanka Ulaga, Tadej Battelino and Urh Groselj

University Medical Centre Ljubljana, University Children’s Hospital, Ljubljana, Slovenia

Introduction: Expanded newborn screening (NBS) is possible with tandem mass spectrometry, a method used for simultaneous testing of many different metabolites, including numerous acylcarnitines and amino acids on dried blood spots (DBS), which enable detection of many aminoacidopathies, organic acidurias and fatty acid oxidation disorders. To prepare an optimal strategy for the organization of the expanded newborn screening for inborn errors of metabolism, a pilot study was performed analyzing 10,048 NBS cards. The expanded NBS was introduced in Slovenia in September 2018. Seventeen metabolic diseases were added to the pre-existing screening panel for congenital hypothyroidism and phenylketonuria, and the newborn screening program was substantially reorganized and upgraded.

Methods: Tandem mass spectrometry was used for the screening of dried blood spot samples. Next-generation sequencing was introduced for confirmatory testing. Existing heterogeneous hospital information systems were connected to the same laboratory information system to allow barcode identification of samples, creating reports, and providing information necessary for interpreting the results.

Results: By the end of 2020 a total of 39,324 samples were screened. Twelve patients were confirmed positive with additional testing. Among them a unusually high incidence of detected patients with Very long-chain acyl-CoA dehydrogenase deficiency was detected.

Conclusions: An expanded newborn screening program was successfully implemented with the first patients diagnosed before severe clinical consequences.

P30. **Retrograde Dried Blood Spot Screening for Tyrosinemia Type 1 in Two Patients**

Jaka Šikonja, Barbka Repič Lampret, Jernej Brecelj, Žiga Iztok Remec, Ana Drole Torkar, Mojca Žerjav Tanšek, Tadej Battelino and Urh Grošelj

Faculty of Medicine, University of Ljubljana, Ljubljana, Slovenia

Tyrosinemia type 1 (Tyr1) is an inborn error of tyrosine (Tyr) catabolism which can cause severe liver dysfunction. Most patients present with clinical signs by age three. Timely treatment can be achieved by NBS primarily using succinylacetone (SA) from DBS. NBS-diagnosis is associated with a more favorable outcome with less disease complications compared to a diagnosis after presentation of symptoms. However, in Europe, NBS for Tyr1 is currently being performed in less than half of the countries.

We aimed to perform a retrograde screening in two patients with Tyr1 using DBS taken at birth (both were born prior to the implementation of expanded NBS program using tandem mass spectrometry in 2018 which has included Tyr1). Analysis was done 3.1 and 5.2 years from DBS collection.

Both patients had elevated SA levels (1.31 and 2.72 μmol/L; cut-off value 0.30 μmol/L) and increased SA to methionine ratio (0.279 and 1.755; cut-off value 0.086); on the other hand, Tyr was elevated only at one patient (364 and 239 μmol/L; cut-off value 320 μmol/L).

We showed that a retrograde diagnosis of Tyr1 was possible after more than three years of birth with SA as a primary marker, complemented by Tyr and SA/methionine ratio. Tyr was less sensitive and normal levels can be seen in Tyr1 patients. NBS for Tyr1 could have resulted in a timely diagnosis and prevented a liver transplantation at the second patient.

Keywords: Tyrosinemia; Newborn Screening; Dried Blood Spot; Retrograde Analysis; Succinylacetone; Tyrosine

P31. **Clinical Presentation, Molecular Analysis and Follow-Up of Patients with Mut Methylmalonic Acidemia in Shandong Province, China**

Xiao Han and Bingjuan Han

Jinan Central Hospital Cheeloo College of Medicine, Pediatrics, Jinan, China

Background: The mut methylmalonic acidemia (MMA) caused by the deficiency of methylmalonyl-CoA mutase (MCM) activity, which results from defects in the MUT gene. The aim of this study was to summarize the clinical and biochemical data, spectrum of mutations, treatment regime and follow-up of patients with mut MMA from Jan 2013 to Dec 2017 in Shandong province, China.

Methods: Twenty patients were diagnosed with isolated mut MMA by elevated C3, C3/C2, and urine methylmalonic acid levels without hyperhomocysteinemia. The MUT gene was amplified and sequenced. Most patients received treatment with specific medical nutrition and oral L-carnitine after diagnosis. Metabolic parameters, clinical presentation and mental development were followed up.

Results: Among 20 patients with mut MMA, 14 had clinical presentations, and 12 presented in the neonatal period. Three patients died of metabolic crises triggered by infection. Twenty-three different mutations were detected, and four mutations (c.613G>A, c.446A>G, c.920-923delTCTT and c.1359delT) were novel. Most patients received timely treatment and had favorable metabolic responses, with reductions in C3, C3/C2 and urine MMA. We obtained 16 records of DQ/IQ assessments. Six patients exhibited normal development, but ten patients suffered from neurological symptoms of varying degrees and had low DQ/IQ scores.

Keywords: follow-up; isolated methylmalonic acidemia; methylmalonyl-CoA mutase; MUT

### 3.7. Spinal Muscular Atrophy

P32. **Spinal Muscular Atrophy (SMA): Screen at Birth, Save Lives**

Marie-Christine Ouillade

SMA Europe e.V., Patient Advocacy, Bordeaux, France

In August 2020, the European Alliance for Newborn Screening in SMA was formed with the objective to reduce the time it takes for a baby born with SMA to be diagnosed. In March 2021, the Alliance launched the Whitepaper: Spinal muscular atrophy: Screen at birth, save lives. We will summarize the highlights of the Whitepaper and add the patient and family perspective to advocating for expanded newborn screening to include SMA. 

In the UN Convention on the Rights of the Child—which has been ratified by all European countries—Article 24 refers to the right to have optimal health care. Newborn screening can help to point to these children that are in particular need for specialized health care. Vice versa, withholding newborn screening from children, however, translates into depriving them of an optimal care pathway. Newborn screening for SMA should be available for all babies in Europe.

We will review how SMA fits the Wilson and Jungner criteria while also highlighting an SMA newborn screening process proposal, the ethical arguments, health economics, and the current landscape of SMA pilot trials in Europe.

P33. **Creating an Alliance to Advocate for Spinal Muscular Atrophy Newborn Screening**

Marie-Christine Ouillade and Robert Pleticha

SMA Europe e.V., Newborn Screening Working Group/Alliance for SMA NBS, Bordeaux, France

The European patient umbrella organization, SMA Europe, created the European Alliance for Newborn Screening in SMA in 2020 to support one of its key initiatives to accelerate newborn screening implementation. The Alliance is made up of SMA Europe’s 23 member organizations from 22 countries along with TREAT-NMD, EURORDIS, EAMDA, and the companies Biogen, Novartis Gene Therapies, LaCar MDX Technologies, PerkinElmer, and Roche. 

The main objective of the Alliance and its members is to reduce the time it takes for a child born with SMA to be diagnosed. To support that objective, the Alliance published a whitepaper in 2021 called Spinal muscular atrophy: screen at birth, save lives. The whitepaper was written with the Alliance Steering Committee, admedicum, and the SMA Europe Working Group on Newborn Screening. Outside experts were involved in the sections on health technology assessment and the newborn screening process in public health systems. The writing and dissemination process was financially supported by a multi-stakeholder funding circle in full compliance with the principles of independence and transparency.

This paper is organized according to the Wilson and Jungner criteria used to judge whether a disease should be included in the newborn screening panel. Since SMA newborn screening meets all the established criteria, newborn screening for SMA should be made available for all babies born in Europe. Detecting and treating 5q SMA early leads to a better clinical outcome for the babies and helps reduce the burden of care for their families.

The European Alliance for Newborn Screening in Spinal Muscular Atrophy demands that national governments and authorities in Europe immediately include a test for spinal muscular atrophy for all newborn children in national newborn screening programs. There is no more time to waste for babies born with SMA to start adequate treatment.

P34. **Newborn Screening for Spinal Muscular Atrophy in the World Today**

Tamara Dangouloff, Eva Vrščaj, Laurent Servais and Damjan Osredkar

Department of Pediatric, University of Liege, Liege, Belgium

Spinal muscular atrophy (SMA) has undergone an unprecedented change since the approval of three disease-modifying therapies. All of these therapies have shown a much more dramatic effect when administered before the first symptoms appear. In this context, newborn screening programs have been set up in several countries.

We conducted a study to understand the status of SMA NBS around the world, the actual population covered, the various processes used, consent requirements, funding, current status-pilot, or official-and projections for the country or region.

We contacted 152 experts around the world and obtained responses from 87 of them. The countries for which we gathered information include 57% of newborns worldwide. We asked the experts what treatments were available in their country and whether newborn screening for SMA was already in place, in a pilot or formal program, or planned. Nine countries were then identified as having NBS; two as an official program—Taiwan and USA—and seven as a pilot or regional project: Austria, Belgium, Canada, Germany, Italy, Japan, and Russia. On 1 January 2021, about 3,674,277 babies worldwide—about 2% of the world population—were already screened for SMA and 288 of them were identified and diagnosed with SMA. The need to expand the SMA NBS was widely expressed. It was noted that having a cost-benefit analysis (68%), health and economic data (52%), and long-term follow-up data on the treatment of pre-symptomatic patients (53%) would be particularly helpful. NBS for SMA is still not widespread in the world today. Our study showed that professionals are all eager to start this NBS. Many obstacles still remain (political, financial) and the example of countries that have developed NBS can be a help for others.

P35. **Newborn Screening for SMA in Southern Belgium: From a Three-Year Pilot Effort to the Official Program**

Tamara Dangouloff, François Boemer, Jean-Hubert Caberg and Laurent Servais

Department of Pediatrics, University of Liege, Liege, Belgium

Spinal muscular atrophy (SMA) is a neuromuscular disorder characterized by progressive muscle atrophy caused by motoneuron death. In its most severe form, without treatment, 90% of children die before the age of two. Since 2016, three treatments have been progressively approved by the FDA and the EMA. The effect of these treatments is much more dramatic when administered before- rather than after- the symptoms. Consequently, newborn screening programs have been initiated in several countries. In 2018, we launched a three-year pilot program to screen newborns for SMA in the Region of Liege, Belgium. This program expended within nine months to whole Southern Belgium (55,000 yearly newborns). In May 2021, 150,202 neonates were screened and eleven were identified with SMA: four with two copies of SMN2 (the most severe form), three with 3 copies (intermediate form) and four with 4 copies (the least severe form). We observed no false positive or negative. One patient with a heterozygous deletion and a point mutation was identified during the course of the program.

Over the duration of the pilot, we progressively improve the processes to decrease turn-around time for results from 15.3 days after birth in March 2018 to 6.5 days in December 2020. 

A strong partnership with the health authorities throughout the pilot program and the involvement of all stakeholders at every stage allowed for a natural transition to a formal program after three years. The involvement of patient advocacy groups, neuromuscular reference centers, and newborn screening centers, as well as public involvement through social and mainstream media also significantly facilitated the rapid and smooth transition to an official program.

P36. **Newborn Screening for Spinal Muscular Atrophy: First Results of a Pilot Study in Latvia**

Madara Kreile, Jekaterina Isakova, Aleksejs Isakovs, Maija Konika, Ieva Micule, Mikus Diriks and Linda Gailite

Scientific Laboratory of Molecular Genetics, Riga Stradins University, Riga, Latvia

Spinal muscular atrophy (SMA) is a heritable neuromuscular disorder that causes degeneration of the alpha motor neurons. Most commonly it is caused by a deletion in the gene SMN1 (Survival of Motor Neuron 1). New disease-modifying treatments have recently been approved and early treatment has been related to a better clinical outcome. In this context, newborn screening for SMA needs to be implemented to ensure early diagnosis of patients. 

The aim of the study is to determine feasibility and utility of NBS for SMA in Latvia.

Methods. From February 2021 till July 2021 4600 parents agreed to participate in the study and were followed with procedure applicable in NBS. SMN1 exon homozygous deletion is detected using qPCR with fluorescent LNA primers, either CFTR exon 17 or gene PRP30 are used as a reference gene for detection of the presence of an intact SMN1 gene or its homozygous deletion. 

Results. In the first month, result delivery time were up to 17 days after sample arrival in the lab., After the approbation of the procedures the median report time is 4 ± 2.4 days. In 14 cases, due to quality of punch or manual mistakes DNA isolation had to be repeated due to poor quality of isolated DNA. In two patients (0.043%) SMN1 homozygous deletion were identified, confirmed later with MLPA method.

Conclusions: The tested sample size is too little to calculate SMA incidence and the project should be continued to ensure statistically significant results.

In a case when the NBS sample is taken within 48–72 h, and it is transported to the lab within two days after sample collection according to legal regulation, positive test results can be delivered to health care professionals before 10th day of life.

Introduction of our method in expanded newborn screening in Latvia is feasible and could facilitate deployment of screening, allowing for early diagnosis that is important for effective treatment.

We suggest that SMA be considered for addition to national recommended uniform screening panel.

P37. **Suitability of Newborn Screening for Spinal Muscular Atrophy in Catalonia (Spain) with the TREC KREC SMA Newborn Screening Kit (Roche Diagnostics^®^)**

Argudo-Ramírez A, Pérez-Fernández J, González de Aledo-Castillo JM, López-Galera RM, Marín-Soria JL, Pajares-García S, Paredes-Fuentes A, Ribes A, Tizzano E, Macaya A, Munell F, Jiménez-Ruiz W and García-Villoria J.

Hospital Clinic, Inborn Errors of Metabolism, Barcelona, Spain

Introduction: Spinal muscular atrophy (SMA) screening was added to the Recommended Uniform Screening Panel (RUSP) from USA in 2018. The benefits of early treatment of SMA make newborn screening (NBS) indispensable. NBS for SMA is performed by detecting the absence of exon 7 in the SMN1 gene by a real-time PCR assay in DNA extracted from dried blood spot (DBS, 3.2 mm) samples. Here, we verify the suitability of the TREC KREC SMA Newborn Screening kit (Roche Diagnostics) for SMA NBS.

Materials and methods: From January to June 2021, we conducted a retrospective SMA screening study with archived DBS samples (August 2020–January 2021) from 3914 newborns using a residual DBS from Catalonian NBS program. The system includes samples punching (BSD 600), the automatic sample preparation system (Hamilton) and the TREC KREC SMA Newborn Screening Kit by real-time PCR (LightCycler^®^480).

We performed the assays with a SMA-positive patient’ sample as internal control in each 96-well plate (not commercially provided). The method includes the analysis for Beta-actin gene as internal quality control in each sample. For the study, we also processed samples in duplicate from five patients with genetically confirmed SMA.

Results: All DBS from Catalonian newborns tested negative for SMA; of note, no type 1 SMA had been clinically diagnosed in any of the infants—aged 5 months or older—By the end of the study. The system showed sensitivity and specificity of 100%. The retest rate was 2% for SMN1 poor amplification and 0.66% for Beta-actin gene poor amplification. Negative results were obtained in all retests.

Conclusions: Our results indicate that Roche Diagnostics^®^ system can be reliably used in SMA newborn screening. A positive SMA control should commercially be provided. To the best of our knowledge, no cases of type 1 SMA have been missed in our screened cohort.

### 3.8. New Disorders

P38. **Newborn Screening of CblC Deficiency: The Importance of Carrying out Second Tier-Tests in the Second Dried Blood Spot**

Silvia Santagata, Emanuele Di Carlo, Teresa Giovanniello, Francesca Nardecchia, Antonio Angleoni and Claudia Carducci

AOU Policlinico Umberto I, Clinical Pathology Unit, Rome, Italy

Background: Defect of cobalamin C (CblC) is an autosomal recessive inherited disease caused by mutation in MMACHC gene (OMIM#609831). This condition is associated with an increase of propionylcarnitine (C3) in dried blood spot (DBS) detected through tandem mass spectrometry and it is included in the expanded newborn screening (NBS) program in several countries. To improve test specificity and positive predictive value (PPV), the increase of C3 is followed by measurement of methylmalonic acid (MMA) in DBS as second-tier test (STT). In the case of positivity, a second DBS is requested. Our aim was to assess the importance to determine MMA in the second sample.

Methods: Our study included newborns examined in the period from 2010 to 2020 for which a second DBS was requested because of alterations of C3 and MMA levels on the first DBS. MMA analysis was performed in the second DBS even if C3, C3/C0 C3/C2 and C3/Met levels were within normal values. 

Results: Nine cases of CblC deficiency were found in Lazio Region during NBS program since 2010. We found that for 5 out of 9 newborns (age 6–20 days) C3 values was above cut-off (3.13 µmol/L) ranging from 5.1–8.5 µmol/L in the second sample. In these newborns, MMA levels were higher than the cutoff (1.37 µmol/L) ranging from 7.75 to 59 µmol/L. In the remaining four CblC newborns, C3 levels evaluated in the second DBS (age 8–20 days) were within normal values (mean 1.79 µmol/L; range: 1.44–2.19 µmol/L), as well as carnitine levels (mean 25 µmol/L, range 21–33 µmol/L) whereas the MMA levels were above the cut-off, ranging from 3.15 to 7.46 µmol/L.

Discussion: Our data demonstrated the importance of performing the analysis of MMA in the second DBS for the detection of CblC deficiency even if C3, C3/C2, C3/C0, and C3/Met levels fall in a normal range. In fact, although C3, C3/C2, C3/C0, and C3/Met are good markers in the first sample, it could fail to identify CblC deficiency in the second sample.

P39. **High Incidence of Neonatal Vitamin B12 Deficiency in Catalonia: Benefits of Newborn Screening Program**

Sonia Pajares, Jose Manuel González de Aledo-Castillo, Rosa María López, Ana Argudo-Ramírez, Aleix Navarro-Sastre, Jose Luís Marín, Frederic Tort, Laura Gort, Jose Antonio Arranz, Clara Carnicer, Aida Ormazabal, Rafael Artuch, Mireia del Toro, Ángeles García-Cazorla, Silvia María Meavilla, Mariela Mercedes de los Santos, Camila García-Volpe, Rosa Fernández, Judit García-Villoria and Antonia Ribes

Hospital Clinic of Barcelona, Section of Inborn Errors of Metabolism-IBC, Department of Biochemistry and Molec, Barcelona, Spain

Introduction: Acquired vitamin B12 (vit-B12) deficiency can result in anemia, failure to thrive, developmental regression and even irreversible neurologic damage if the deficiency is prolonged and untreated. Early detection allows starting a prompt treatment preventing severe complications. In recent years, acquired neonatal vit-B12 deficiencies were described by several Newborn Screening Programs (NBSP). Based on this evidence, we wanted to evaluate the effectiveness of our screening strategy in the detection of this condition in our program.

Methods: Our study included 403,252 newborns analyzed from 2013 to 2018. From 2013 to 2014, our initial strategy was to ask for a second dried blood spot (DBS) to reanalyze the primary markers and dried urine spot to analyze organic acids. Since 2015, our strategy was the analysis of second tier-tests (2TT) (homocysteine (Hcys), methylmalonic acid (MMA) and methylcitric acid (MCA)) on the first DBS.

Results: 144 newborns with vit-B12 deficiency were detected resulting in a high incidence in our population (1:2800), higher than previous descriptions. Concerning primary markers, C3 was the most sensitive (81%) followed by C3/C2 (22%) and C3/Met (17%). Moreover, among 2TT, Hcys was the most sensitive (87%) followed by MMA (60%). The inclusion of 2TT in our NBSP allowed decreasing the cut-off of primary markers, increasing the sensitivity five times with respect to the first strategy; to reduce drastically the recall rate in 93% and, to avoid false negative results. Vit-B12 was low (<198 pmol/L) in 106 newborns (72% due to maternal vit-B12 deficiency) and 38 were classified as functionally deficient. Patients were treated with 1 mg i.m. hydroxocobalamin. Based on this evidence, the Autonomous Government of Catalonia recommended avoiding low vit-B12 ingestion during pregnancy.

Conclusions: The inclusion of 2TT in our NBSP was successful in detecting acquired vit-B12 deficiency. We included the screening of this condition in our NBSP, since its detection has potential benefits.

P40. **Health Outcomes of Infants with Vitamin B12 Deficiency Identified by Newborn Screening and Early Treated**

Ulrike Mütze, Gwendolyn Gramer, Sven F. Garbade, Florian Gleich, Dorothea Haas, Sarah C. Grünert, Julia B. Hennermann, Eva Thimm, Junmin Fang-Hoffmann, Jürgen G. Okun, Georg Hoffmann and Stefan Kölker

Heidelberg University Hospital, Stoffwechselzentrum, Zentrum für Kinder- und Jugendmedizin, Klinik 1, Heidelberg, Germany

Background: Newborn screening (NBS) for vitamin B12 deficiency was shown to be feasible, identifying an unexpectedly high birth prevalence between 3–19 in 100,000 (i.e., 1 in 30,000–5355) newborns. The aim of this study was to evaluate the clinical and cognitive outcomes at age 1.5 ± 0.5 years of infants with vitamin B12 deficiency identified by NBS and early treated.

Methods: A prospective multi-center observational study on health outcomes of 31 infants with vitamin B12 deficiency identified by NBS. Neurodevelopment was assessed by Denver Developmental Screening Test (DDST).

Results: In 285,862 newborns screened between 2016 to 2019, the estimated birth prevalence of vitamin B12 deficiency was 26 in 100,000 newborns, with high seasonal variations (lowest in summer: 8 in 100,000). Infants participating in the outcome study (N = 31) were supplemented with vitamin B12 for a median (range) of 5.9 (1.1–16.2) months. All achieved age-appropriate test results in DDST at age 15 (11–23) months and did not present with symptoms characteristic for vitamin B12 deficiency. Most (81%, N = 25) mothers of affected newborns had a hitherto undiagnosed (functional) vitamin B12 deficiency, and, subsequently, received specific therapy. 

Conclusions: Neonatal vitamin B12 deficiency can be screened by NBS, preventing the manifestation of irreversible neurological symptoms and the recurrence of vitamin B12 deficiency in future pregnancies through adequate treatment of affected newborns and their mothers. The high frequency of mothers with migrant background who have a newborn with vitamin B12 deficiency highlights the need for improved prenatal care.

P41. **3-O-Methyldopa and 5-Hydroxytryptophan Assay in Dried Blood Spot by Ultra-Performance Liquid Chromatography Tandem Mass Spectrometry: A Useful Tool for the Screening of L-Amino Acid Decarboxylase Deficiency**

Emanuele Di Carlo, Silvia Santagata, Luca Sauro, Manuela Tolve, Filippo Manti, Vincenzo Leuzzi, Antonio Angeloni and Claudia Carducci

Department of Experimental Medicine, Sapienza University of Rome, Rome, Italy

Background and aims: Aromatic L-amino acid decarboxylase (AADC) deficiency is a rare inherited disorder of neurotransmitter synthesis presenting with early onset encephalopathy, which results in disabling neurological impairment. The importance of early diagnosis and treatment has recently been highlighted by new treatment options, such as gene therapy. In the last few years, pilot newborn screening programs for AADC deficiency, based on 3OMD determination in DBS, were activated. We developed a specific diagnostic tool to detect 3-O-methyldopa (3OMD) and 5-hydroxytryptophan (5HTP), two markers of AADC deficiency, in dried blood spot (DBS).

Materials and methods: After extraction from DBS, 3OMD and 5HTP were analyzed by ultra-performance liquid chromatography and tandem mass spectrometry. Instrument parameters were optimized to obtain the best sensitivity and accuracy.

Results: Chromatographic separation was accomplished in 13 min. The limit of detection was 2.4 and 1.4 nmol/L of blood for 3OMD and 5HTP respectively, and response was linear over the range 25–5000 nmol/L. Between-run imprecision was <9% (3OMD) and <13% (5HTP). Owing to dynamic 3OMD age-dependent decline, an age-specific continuous reference range was established. DBS samples from four patients with AADC deficiency showed a marked increase of 3OMD and 5HTP.

Conclusion: Simultaneous measurement of 5HTP and 3OMD in DBS improves diagnostic test specificity and sensitivity. The developed method could be used as second tier test in screening programs for the early diagnosis of AADC deficiency as well as in diagnostics workup of symptomatic patients.

P42. **Maternal Spot Urinary Iodine and Creatinine Corrected Urinary Iodine with Follow Up of Maternal and Neonatal Thyroid Functions Outcomes as Dyads—A Prospective Pilot Research Study from India at a Tertiary Care Centre in Chennai**

Sudha Rathna Prabhu and Rathina Easwar

The Tamil Nadu Dr MGR Medical University, Chennai (Tamil Nadu), India

Objectives: To estimate spot urinary iodine concentrations (SUIC) creatinine corrected urinary iodine concentrations (CUIC) in pregnant women in third trimester, follow up maternal and neonatal thyroid outcomes as dyads and assess iodine adequacy in salt used during pregnancy. Methods: The study is part of PhD Research at The Tamil Nadu Dr MGR Medical University Chennai. Urinary iodine was estimated by ammonium persulphate method. In pregnancy, the WHO cut off below 150 µg/L was defined as iodine deficiency. Neonatal TSH was done by DELFIA in dried blood spots with heel prick samples collected after 72 h. The ECLIA method was used for serum TSH and free thyroxine. All tests were done at accredited medical lab SVASAM Health Care Centre with EQUIP, CDC Atlanta NBSQAP enrollments. 

Design: This was an observational, prospective study. Study site: Joseph Hospital. Inclusion Criteria: Normal pregnant women at 34 to 36wks as singleton pregnancies. Exclusion Criteria: Known thyroid disorders. Study number: 147 mother-baby dyads. Statistics: Descriptive with frequency, percentage, mean for normal and median for skewed data. Outcomes: SUIC and CUIC were below 150 µg in 13.6% and 5.1% pregnant women. Chi-square test showed significant p value below 0.05 for SUIC and CUIC with maternal TSH and neonatal TSH values. Estimated prevalence of iodine deficiency was 18.38% as SUIC and 13.92% as CUIC. Maternal hypothyroidism was newly detected in 18.4% participant; 98.5% used salt with iodine less than 15 ppm tested with UNICEF rapid test kits. Mean neonatal birth weight was 3034 g. Low birth weight 5.4%. Preterm births were 1.4%. One neonate was screened as positive for congenital hypothyroidism. Neonatal TSH values were above 5 µIU/L in 17.7% and as per WHO criteria indicate iodine deficiency in study population. 

Conclusion: This was a pilot study on maternal and neonatal dyads indicate iodine deficiency in pregnant women warranting more regional studies to recommend public health strategies.

### 3.9. New Methodology and Quality Improvement

P43. **Optimizing Phenylalanine Cut-Off Levels in Newborn Screening Program**

Daša Perko, Barbka Repič Lampret, Žiga Iztok Remec, Vanja Čuk, Blanka Ulaga, Domen Trampuž, Blaž Krhin, Adrijana Oblak, Ajda Biček, Mojca Žerjav Tanšek, Ana Drole Torkar and Urh Grošelj

University Children’s Hospital, UMC Ljubljana, Clinical Institute for Special Laboratory Diagnostics, Ljubljana, Slovenia

In Slovenia, the NBS program for Phenylketonuria was established in 1979. We aimed to analyze DBS samples from larger group of newborns to optimize cut-off levels for PKU screening.

In first part of a study we prospectively included all newborns born from 2018–2020. Cut-off value for phenylalanine (Phe), using fluorimetric method, for at which additional sample was required was 120 µmol/L. Confirmatory analyses included Phe determination in serum and genetic analyses of PAH gene. The final definition of the true screening-positive was made based on genetic result and whether the child required a low-Phe diet. In second part of the study, we retrospectively reviewed data from all patients with hyperphenylalaninemia (HFA) born from 2000–2018 (cut-off was 120 µmol/L) and assessed the safety and sensitivity of elevating the cut-off to above 150 µmol/L.

Of the 37,784 samples (2018–2020), 108 Phe results exceeded 120 µmol/L. After confirmatory analysis, seven samples turned out to be true positive and 101 samples false positive. If we adjusted the cut-off value to 150 µmol/L, number of recalls would be 16 instead of 108, thus false positives would be only nine instead of 101. All the true-positives would be detected. When adjusting cut-off to 180 µmol/L, number of recalls would be 9, but still detecting all true positives.

Of the 380,000 samples (2000–2018), 72 patients were diagnosed with PKU or HFA. If we raised cut-off to 150 µmol/L, all of those patients would have been detected. If we additionally raised cut-off value to 180 µmol/L, we would miss three true-positive patients.

We demonstrated on a large group of newborns that cut-off value at 150 µmol/L instead of 120 µmol/L using fluorometric method is safe, with zero false negatives. Furthermore, raising cut-off makes this method more specific, yielding much lower number of false positives and is thus less burdensome for both parents and newborns.

P44. **Investigating Strategies for Overcoming the Challenges of Low and Non-Homogeneous Biotinidase Activity in QA Specimens**

Elizabeth McCown and Konstantinos Petritis

Centers for Disease Control and Prevention, Division of Laboratory Sciences, Atlanta, GA 30341, USA

Biotinidase (BIO) deficiency is an inherited disorder where the patient’s body is unable to recycle their supply of biotin. In the newborn screening (NBS) community, the BIO enzyme has gained a well-deserved reputation for being notoriously labile. It is not uncommon for NBS laboratories to experience increases in false positives for BIO deficiency during summer months and, more sporadically, when transitioning to new lots of collection cards. As a provider of NBS quality assurance (QA) specimens, the fickle nature of BIO also causes problems for the Newborn Screening Quality Assurance Program (NSQAP) at the CDC. One of the greatest challenges has been achieving homogeneity of BIO enzyme activity in a single lot of DBS QA materials. We have repeatedly seen wide variations in activity from card-to-card—and even from spot-to-spot on a single card—but without any apparent, repeatable pattern. The problem is so significant that it has prevented us from being able to provide suitable quality control (QC) materials for BIO to our participants; currently, we distribute only proficiency testing (PT) materials for BIO. Attempts to identify a cause included investigations of ambient temperature and humidity during drying and efforts to increase air flow around the drying cards. We have recently determined that shortening the drying time (before package and storing) from overnight to three hours has a dramatic positive effect on both overall BIO activity as well as its homogeneity. We are conducting a series of trials to characterize the impact of drying time on BIO and determine the limits we must adhere to for a usable BIO QC product. Defining this time range will determine the maximum size of the pool we are able to create and thus, the number of participants we can accommodate. Having this information will allow us to better serve the NBS community by providing reliable QC materials for BIO for the first time.

P45. **Diagnosing Sickle Cell Disease in Children in Sierra Leone with Sickling Test and SickleSCAN™ Point-of-Care Test: A Case Series**

Stephanie Ibemere, Joseph Edem-Hotah, Joan Shepherd and Emily Thurmond

School of Nursing, Durham, Duke University, Durham, NC, USA

Sickle cell disease (SCD), a hemoglobinopathy of growing global significance, presents significant challenges in Africa, where limited conventional laboratory diagnostics for early diagnosis contribute to high early infant mortality rates. We aimed to demonstrate the feasibility of early diagnosis of SCD in limited-resource settings with a novel point-of-care test (POCT). We carried out a 14-month study in a charity-care community clinic in rural eastern Sierra Leone. We used the SickleSCAN™ POCT and the high-performance liquid chromatography (HPLC) method to confirm SCD in 125 children with positive Emmel test results for sickle cell trait. The SickleSCAN™ identified 48.8% patients with SCD, 42.4% with sickle cell trait, and 11 with normal hemoglobin. The SickleSCAN™ showed discordance with the HPLC method for 2 of 21 samples. Our data supports community-based SCD POCT diagnosis programs where confirmatory laboratory testing is scarce. Future studies should assess barriers to the implementation of community-based SCD POCT diagnosis.

P46. **Outlining the Role of Nurse Champions in Implementing Newborn Screening with Point-of-Care Testing for Early Diagnosis and Care Access for Sickle Cell Disease**

Stephanie Ibemere

School of Nursing, Duke University, Durham, NC, USA

Access to point-of-care (POCT) services has shown potential to alleviate some diagnostic challenges associated with laboratory-based methods in low- and middle-income countries (LMICs). Improving accessibility to POCT services during antenatal and perinatal care is among the global health priorities to improve maternal and child health. This expert lecture provides insights on the availability of POCTs designed for diagnosing sickle cell disease (SCD) in LMICs.

The World Health Organization has recommended implementing SCD newborn screening (NBS) programs to include advocacy, prevention and counseling, early detection, treatment, research, and community education as the way forward in LMICs. This process requires the selection of program implementation strategies that fit local contexts and local champions who have sufficient technical knowledge and experience to guide relevant stakeholders.

We propose that nurses and midwives champion SCD-POCT programs because they can effectively handle case identification, management, and patient education. In many LMICs, the management of SCD devolves to local primary healthcare centers where nurses and midwives primarily provide maternal and child healthcare services. 

The Nurse Champion SCD-POCT Model we propose is a case study exploring the wide variety of nursing roles in the early diagnosis of genetic diseases centered on POCTs. The study conceptual framework is underpinned by Program Science, an implementation science framework. Program Science bridges the disconnect between researchers, clinicians, and policymakers in public health programs. Program Science ensures that research and science are embedded within public health programs and that public health programs drive research questions based on field-level challenges and knowledge gaps. We demonstrate how nursing practice roles could inform research and how nursing research could generate critical scientific contributions to informs practice and policy.

P47. **Dry PCR in Detecting the Absence of SMN1, TREC and KREC from Extracted DNA from DBS**

Terhi Helenius, Henna Savela, Ville Veikkolainen, Mikko Aaltoranta and Mikael Hjort

PerkinElmer, Wallac, Turku, Finland

PerkinElmer has recently developed a ‘for research use only’ dry qPCR assay for the qualitative detection of the homozygous deletion of survival of motor neuron 1 (SMN1) gene exon 7 and the quantification of T-cell receptor excision circles (TREC) and kappa-deleting recombination excision circles (KREC). This assay can run up to 96 samples in less than 2 h from a dried blood spot sample (DBS). The Eonis™ SMN1, TREC, KREC assay uses dried blood spot (DBS) samples and consists of a short 2-step DNA extraction protocol and sample addition to the PCR plate, and therefore does not require any dedicated clean room or freezer space.

The Eonis™ SMN1, TREC, KREC kit detects qualitatively the absence of exon 7 in the SMN1 gene simultaneously with the quantitative detection TREC and KREC copy numbers. The multiplex assay uses ribonuclease P/MRP subunit P30 (RPP30) as an internal amplification control, to monitor the quality of the extracted DNA. The assay is monitored using SMN1/TREC/KREC-positive and SMN1/TREC/KREC-negative DBS controls, which are extracted and processed simultaneously with the samples throughout the whole workflow. The Eonis™ SMN1, TREC, KREC kit is developed together with the Eonis™ Q PCR cycler, making up a robust, reliable, and easy to use total solution consisting of both reagents and instruments.

Regardless of research lab size and resourcing, the Eonis™ SMN1, TREC, KREC kit is an elegant, simple, and reliable research method for the detection of SMN1, TREC and KREC absence in extracted DNA. Please check with your local representative for availability.

P48. **Heads Up: A Technical Approach/Study to Prepare Challenges Ahead. Towards Next-Generation Sequencing (NGS)-Based Newborn Screening**

Abigail Veldman, Rebecca Heiner-Fokkema, Marcel Nelen, Richard Sinke, Peter Schielen and Francjan van Spronsen

University of Groningen, University Medical Centre Groningen, Division of Metabolic Diseases, Beatrix Children’s Hospital, Groningen, The Netherlands

Introduction: Newborn screening (NBS) aims to identify neonates with a life-threatening condition for which immediate treatment is life-saving or significantly reduces morbidity. Currently, a biochemistry-first approach is used for inherited metabolic disorders (IMD) and other (inborn) disorders. Next-generation sequencing (NGS) allows detecting IMDs lacking an identifiable biochemical footprint. We designed a technical study to gradually explore NGS-first techniques for NBS (NGSf4NBS). In this study, the NGSf4NBS project and associated thoughts and challenges are described. 

Methods: In three WorkPackages (WPs) we will explore NGSf4NBS. WP1 aims to identify a list of IEM eligible for NGS-first testing based on treatability. WP2 aims to develop a rapid NGS-based workflow for NBS establishing feasibility, limitations and perform a comparison of different technical approaches. Finally, WP3 will prepare for an implementation study toward the incorporation of this workflow in the existing NBS program and propose an action plan for positive NGS-test results.

Results: We report on the selection process performed in WP1 and the resulting list of eligible IMDs. Secondly, we report on a comparison between various types of NGS in WP2. Thirdly, we present an elaborate strategic plan for WP3 in which foreseen challenges around implementation will be addressed.

Discussion/Conclusion: The results of this study may be the start of an additional analytic route within NBS strengthening current, biochemistry-driven NBS. Still, numerous challenges have to be dealt with, e.g., defining treatability, proving the accuracy of NGSf4NBS, dealing with genetic variances of unknown significance and possible second-tier testing, and technicalities around implementing NGS on an (inter)national scale.

P49. **Experiences with the Rapid Screening for Hereditary Metabolic Diseases (RuSH) Protocol**

Emmalie Jager, Charlotte Lubout, Andrea Schreuder, Ronald Maatman, Francjan van Spronsen, Rebecca Heiner-Fokkema and Terry Derks

Beatrix Children’s Hospital, University Medical Center Groningen, Metabolic Diseases, Groningen, The Netherlands

Introduction: With the neonatal RusH protocol we aimed to prevent early symptomatology and provide screening results within the first week of life in neonates with a known family member with a hereditary metabolic disease (HMD), or mother with acute fatty liver in pregnancy (AFLP). This is done by instituting feeding regimens and dried blood spots (DBS) analysis of cord-blood and heel sticks before second feeding and 24 h after birth. 

Methods: We analyzed six years of experience with the RuSH protocol, based on DBS acylcarnitine and amino acid profiles and clinical outcomes. Sensitivity, specificity, positive and negative predictive values were calculated for all moments of blood sampling in 31 neonates (25 families) with phenylketonuria, various fatty acid oxidation disorders, riboflavin transporter deficiency, and AFPL.

Results: Positive and negative predictive values for sampling at birth, just before second feeding at day 0, around 24 h after birth were (positive) 0.56, 0.5, 0.5, and (negative) 1, 1, 1, respectively for all indications. RuSH screening results were medially available on the second day of life. No severe symptoms were recorded.

Discussion/conclusion: Exclusion and suspicion of an HMD is possible and feasible rapidly after birth. Subsequent measurements enabled metabolic profile interpretation.

P50. **A Novel Algorithm to Facilitate the Inclusion of Inherited Metabolic Diseases on Newborn Screening Programmes**

David Cheillan, Alberto Burlina, Heather Church, Simon Heales, Teresa Hoi Yee Wu, Patricia Roberts, Georgina Morton, Anupam Chakrapani and Erica Sluys

Groupement Hospitalier Est-Hospices Civils de Lyon, Service Biochimie et Biologie Moleculaire, Lyon, France

Despite the rich discourse on how to come together across Europe and harmonize newborn screening (NBS) programs (Loeber et al., 2021, Cornel et al., 2011, Castineras et al., 2019), there has been little forward motion. Most European countries follow the World Health Organization criteria to determine which disorders are appropriate for screening at birth; however, these criteria are interpreted and implemented by individual countries differently, creating disparities. A novel and robust algorithm was built to objectively assess and prioritize IMDs for inclusion on expanded NBS programs. The Wilson and Jungner classic screening principles (Wilson and Jungner 1968) were used as a foundation, and the algorithm is structured upon three pillars: treatment, diagnosis and natural history of the disorder. The proposed algorithm is a point-based system composed of individual and measurable criteria, designed to objectively evaluate and prioritize inherited disorders. The algorithm does not include a cost effectiveness analysis, but instead facilitates the identification of disorders that would then need to be assessed in a health economics analysis phase. Our goal is for this algorithm to pave the way forward for evidence-based expansion of NBS programs, by allowing countries to objectively evaluate disorders while maintaining the ability to separately evaluate specific economic, societal and political aspects of their own screening programs. The proposed algorithm could limit the room for interpretation on which IMDs could be added to NBS programs, reduce the disparity across European countries, and allow for horizon scanning of disorders for future consideration.

P51. **Development and Validation of Multiplexed Second-Tier Newborn Screening Assays**

Matthew Kilgore, Samantha Isenberg, Edgardo Lobo, Dimitrios Platis, Austin Pickens and Konstantinos Petritis

Centers for Disease Control and Prevention, Newborn Screening and Molecular Biology Branch, Atlanta, GA, USA

Second-tier newborn screening analyses are applied to increase the specificity of a positive first-tier screening assay in many public health laboratories. One drawback of current second-tier screening approaches is they often rely on disorder-specific LC-MS/MS methods, requiring the maintenance of separate second-tier methods and instrumentation systems. These assays are also prone to delays from specimen batching. To address these challenges, we have been developing and validating a multiplexed hydrophilic interaction liquid chromatography (HILIC) LC-MS/MS method capable of simultaneously detecting multiple biomarkers indicating inborn errors of metabolism. The HILIC method separates non-derivatized biomarkers for maple syrup urine disease, homocystinuria, methylmalonic acidemia, propionic acidemia, short-chain acyl-CoA dehydrogenase deficiency, guanidinoacetate methyltransferase deficiency, glutaric acidemia type 1, glutaric acidemia type II, X-linked adrenoleukodystrophy, and Pompe disease from known interferents. When developing the second-tier HILIC method for optimal sample extraction and chromatography, extraction solvent, mobile phase composition, and gradient timing were all important. For the second-tier HILIC method, validation, precision, linearity, limit of quantification, specificity, carryover, reproducibility, and accuracy were evaluated. We are also developing a multiplexed reversed phase (RP) LC-MS/MS method. The RP assay uses a derivatization step before analyzing biomarkers of maple syrup urine disease, homocystinuria, methylmalonic acidemia, propionic acidemia, short-chain acyl-CoA dehydrogenase deficiency, guanidinoacetate methyltransferase deficiency, and Pompe disease. These highly multiplexed HILIC and RP-based LC-MS/MS methods show promise for streamlining second-tier newborn screening tests in public health laboratories to simplify workflows and optimize resource use.

P52. **Important Lessons on Instability of Amino Acids in Dried Blood Spots**

Allysa Dijkstra, Pim de Blaauw, Willemijn van Rijt, Hanneke Renting, Ronald Maatman, Francjan van Spronsen, Peter Schielen, Terry Derks and Rebecca Heiner-Fokkema

Laboratory Medicine, Metabolic Diseases, University Medical Center Groningen, University of Groningen, Groningen, The Netherlands

Introduction: Stored heel prick Dried Blood Spots (DBS) are valuable samples for retrospective investigation of inborn metabolic diseases (IMD), including post-mortem investigations, biomarker evaluation and validation studies. However, many metabolites suffer degradation during long-term storage. Therefore, we investigated the 5-year stability of amino acids in stored heel prick DBS.

Methods: This study used data from the retrospective BURDEN study, which aimed to investigate IMD prevalence in deceased children. Amino acid profiles were analyzed in 2018 using a validated LC-MS/MS method in 2170 anonymous heel prick DBS stored from 2013 to 2017. Stability was assessed by calculating amino acid changes over the years and by Jonckheere’s-Terpstra trend tests. DBS were stored at +40 °C during the first year and at room temperature thereafter at the Dutch National Institute of Public Health and the Environment (RIVM). Non-parametric outlier tests were used for outlier removal.

Results: From least to most stable, concentrations of glutamine, tryptophan, taurine, lysine, asparagine, glycine, ornithine, serine, threonine, phenylalanine, tyrosine, leucine, valine, and isoleucine decreased significantly over five years of storage. Concentrations of hydroxyproline, citrulline and aspartate remained stable, while concentrations of alanine, glutamate and proline remained stable for up to two years.

Conclusion: Instability of amino acids may cause incorrect interpretation of test results from stored DBS. This can greatly impact retrospective biomarker studies and IMD diagnostics. For these purposes, adequate storage of DBS, preferably at −80 °C under humidity-controlled conditions, is of utmost importance. If this cannot be achieved, use of control DBS collected under similar storage conditions is necessary.

P53. **SPOT-itTM Screening Assay results**

Diogo Silva

ImmunoIVD, R&D, Nacka Strand, Sweden

If left untreated, Spinal Muscular Atrophy (SMA) and Severe Combined Immunodeficiency (SCID) can be fatal genetic conditions in newborns, but the recent release of new treatments is improving lives of patients. In both cases, evidence shows that early diagnosis leads to more effective treatment and better outcomes. Newborn screening offers the option of diagnosis soon after birth and before symptom onset.

The newly developed ImmunoIVD SPOT-itTM TREC, KREC & SMN1 Screening Kit was used to analyze samples from European newborns, including known SMA and SCID positive samples. The Screening Kit uses sensitive qPCR technology to determine copy numbers for.

TREC and KREC and Ct-values for SMN1 markers and includes quantification of the endogenous control Beta-Actin (ACTB) in every well as a control for DNA amplification. Samples were run according to the included flowchart—initially singly and then rerun in duplicate if either the TREC, KREC or SMN1 markers were below a predetermined cutoff.

Samples were classified as ‘screening positive’, ‘negative’, or ‘inconclusive’ according to the flowchart. Inconclusive results are obtained when both the markers and ACTB had low values, suggesting low DNA quantity or quality. Re-run rate as well as sensitivity and specificity for each disease is presented.

The SCID-SMA Screening assay offers a convenient, accurate and cost-effective way of screening for two disorders in one multiplex reaction.

P54. **Incorporation of Second-Tier Tests and Secondary Biomarkers to Improve the Positive Predictive Value (PPV) Rate in Newborn Screening Program**

Sarang Younesi and Bahreh Yazdani

Nilou Medical Laboratory, Neonatal Screening, Tehran, Iran

Background: Presently neonatal screening has become an essential part of routine newborn care in the world. This Screening is a non-invasive evaluation that includes inborn errors of metabolisms (IEMs) using tandem mass spectrometry (LC-MS/MS).

Participant: This retrospective study was conducted in 39,987 Iranian newborns that were referred into Nilou Medical Laboratory, Tehran, Iran, for newborn screening programs of inborn errors of metabolisms (IEMs). Statistical data were recorded via call interviewing in 6–8 months after their screening tests.

The objective of the study: The main objectives of this study were to obtain the correlation between the screening result and evaluation of screening criteria such as prevalence, positive predictive value (PPV), negative predictive value (NPV), false positive rate (FPR), and recall rate. In addition to we evaluate the rule of second-tier tests and secondary biomarkers for improving the efficacy of newborn screening program.

Result: The mean age of all participants was 3.9 ± 1.1 days; 5.1% of participants were over 13 days and 7.7% were preterm or underweight. A total of 11,384 (29.4%) of the cases came from a consanguineous marriage and the type of delivery in 8332 (51.3%) of valid cases in this issue was due to a cesarean delivery. The neonatal screening results were performed that the overall negative predictive value (NPV) is 100% and the overall positive predictive value is 32.2%. Our study also ≥demonstrated that our FPR is 0.25% and overall prevalence is 1:833.

Discussion and Conclusion: To improve our results, a primary goal is the development of an applying appropriate laboratory’s cut-offs and secondary biomarkers also second-tier test to decrease the false positive rate and to improve the PPV. Indeed, there are lots of second-tier strategies available to reduce false tests. This study shows that due to a high rate of consanguineous and endogamy marriages in Iran incidence of metabolic disease increased.

P55. **Enabling Workflow Efficiency Using a New Generation Dried Blood Spot Punch Instrument with Advanced Data Automation**

Andrew Stewart and Ali Baradaran

BSD Robotics, Engineering, Brendale, Australia

Adoption of more advanced technologies such as molecular methods and the addition of more tests to newborn screening panels are increasing trends. Such advances demand a need for improved system integration and data automation to achieve higher standards of efficiency and information recording. This context includes pre-analytical sample preparation prior to analytical steps where technical advances have significant potential to improve turnaround times and quality of result.

To address this, BSD Robotics has dedicated extensive research and development to a new punch instrument improve pre-analytical processes in newborn screening laboratories. The BSD Galaxy A9 is discussed with relevance to a newborn screening setting, and in particular, molecular assay compatibility and automation integration.

P56. **Opportunities and Challenges of Metabolomics in Newborn Screening**

Austin Pickens, Konstantinos Petritis and Carla Cuthbert

Centers for Disease Control and Prevention, Newborn Screening and Molecular Biology Branch, Chamblee, GA, USA

Newborn screening (NBS) aims to identify asymptomatic newborns at risk of rare diseases such as inborn errors of metabolism (IEM). IEMs most often result in toxic accumulations of metabolites, along with deficiencies in downstream metabolites due to lack of normal enzymatic function. Furthermore, to cope with the metabolic stress of IEMs, elevated metabolites are also shunted to alternate biochemical pathways resulting in unique metabolic products. While traditional NBS has focused on targeting single metabolites for screening, recent trends in the literature demonstrate the decreased false positive rate when using more than one metabolite, metabolite ratios, or using several metabolites to classify presumptive positive samples. Untargeted metabolomic studies on newborn specimens reveal new novel markers able to better distinguish disease phenotypes when used with or in the place of conventional markers. Our group recently reported a novel hybrid targeted and untargeted metabolomic workflow using direct infusion coupled to high resolution mass spectrometry that builds on traditional NBS workflows integrating instrumentation with higher specificity. Here we present a brief history of metabolomics in NBS, recent studies identifying new markers with higher specificity for some IEMs, informatic considerations, our current platform and efforts, and future considerations toward metabolomics in NBS.

P57. **Evaluation of Hematocrit-Independent Devices versus PE 226 DBS in Lysosomal Diseases Screening Using Multiplexed Tandem Mass Spectrometry Assays**

Franklin Ducatez, Carine Pilon, Stéphane Marret, Soumeya Bekri and Abdellah Tebani

Rouen University Hospital, Metabolic Biochemistry & Neonatal Pediatrics, Intensive Care, and Neuropedi, 76,000-Rouen, France

Background: Dried blood spots (DBS) present numerous advantages in newborn screening (NBS) but might suffer from hematocrit effect when analyzing a subpunch or the punch position on the blood spot. This effect could be avoided using hematocrit-independent sampling devices such as the hemaPEN^®^ which collects the blood via integrated microcapillaries, each depositing the blood on a prepunched paper disk. 

Objective: In this study, we compared the technical performance of the prepunched 3.5 mm disks within hemaPEN devices to 3 mm punches issued from PE 226 DBS, including the punch position on the blood spot, using a multiplex-tandem mass spectrometry (MS/MS) enzymatic activity assay of 6 lysosomal enzymes (NeoLSD^®^ assay). 

Methods: Activities of ABG (Gaucher), GAA (Pompe), GLA (Fabry), and IDUA (MPS-I) in DBS were determined by MS/MS using the NeoLSD^®^ assay system. We used three different hematocrit levels (30%, 40–50% and 60%). For each DBS, three punching positions were used (center, intermediary and border). Three replicates were performed for each condition. 

Results: No significant effect was observed regarding the sample hematocrit nor punch position. Hierarchical clustering showed consistent similarity between each sample replicates independently of the experimental conditions. The spearman correlations between HemaPEN prepunch and conventional DBS punch spanned between 0.62 and 0.95 across all assessed hematocrit levels and punch positions. 

Conclusion: Based on these real-world patient data, hematocrit and sampling procedure have no effect on the assessment of enzyme activity using the NeoLSD^®^ assay. These results highlight the reliability of DBS PE 226 for this assay.

P58. **RNA Splicing: A New Paradigm in Multiple Acyl-CoA Dehydrogenase Deficiency (MADD) Patients Identified by Newborn Screening**

Célia Nogueira, Lisbeth Silva, Ana Marcão, Carmen Sousa, Helena Fonseca, Hugo Rocha, Teresa Campos, Elisa Leão Teles, Esmeralda Rodrigues, Patrícia Janeiro, Ana Gaspar and Laura Vilarinho

National Institute of Health Doutor Ricardo Jorge, Human Genetics Department, Porto, Portugal

Multiple acyl-CoA dehydrogenase deficiency (MADD), also known as glutaric acidemia type II, is classically caused by a congenital defect in electron transfer flavoprotein (ETF) or ETF dehydrogenase (ETFDH).

Simultaneous DNA and RNA investigation can increase the number of MADD patients that can be confirmed following suggestive data results of expanded newborn screening program. In clinical practice, accurate identification of pathogenic mutations is fundamental, particularly with regards to diagnostic, prognostic, therapeutic and ethical issues. We focused our study on 4/10 unrelated MADD patients detected through expanded NBS whose incidence in our country is 1:142,204 newborns.

DNA sequencing was used to confirm all patients except four, in which was required a RNA strategy to identify the second causative mutation. These four patients were classified as type II without congenital anomalies. Molecular diagnosis revealed seven mutations in ETFDH gene, being four of them novel variants.

The molecular confirmation of MADD diagnosis in the studied patients was just possible due to the RNA studies carried out, as three of these novel variants affect the splicing process. RNA sequencing allowed the identification of the second causative allele in these patients and the confirmation of this presumptive diagnosis, only based on the neonatal acylcarnitine profile.

Our work highlights the importance of RNA studies for a definitive diagnosis of MADD patients, expands the background of ETFDH mutations and will be important for an accurate genetic counseling and a prenatal diagnosis to the affected families.

P59. **Near-Infrared-Based Hematocrit Prediction of Dried Blood Spots as a Step toward Increased Implementation of Patient-Centric Sampling: An In-Depth Evaluation**

Liesl Heughebaert, Lisa Delahaye, Christoph Lühr, Stijn Lambrecht and Christophe Stove

Ghent University, Bio-Analysis, 9000 Ghent, Belgium

Background: Dried blood spot (DBS) microsampling has attracted interest in different clinical fields, including pediatrics, owing to its many advantages compared to conventional blood sampling. However, while being applied for decades for screening purposes, some challenges, such as the hematocrit (Hct) effect, hinder further widespread use of DBS for quantitative purposes in clinical practice. Among the approaches that were developed to cope with this issue, is the Hct prediction of DBS using near-infrared (NIR) spectroscopy. The aim of this study was to extensively evaluate a commercially available NIR set-up for the prediction of the Hct from DBS.

Methods: Using left-over venous EDTA-anticoagulated blood from patients, the accuracy and precision, stability, and robustness were assessed. Applicability of the method on capillary DBS was evaluated via finger prick samples.

Results: Following actualization of an in-built calibration model, which was needed as an unacceptable negative bias was observed, the method validation resulted in a maximal bias, respectively total precision, of 0.013 L/L and 4.5%. The method was robust toward several aspects, including storage (except for storage at 60 °C), measurement location, type of filter paper (Whatman 903 vs. Ahlström 226) and spotted volume. Furthermore, the method allowed to discern an altered blood spreading in DBS that had been pressed following collection. In contrast, holding the filter paper at an angle of approximately 45° while collecting the DBS did not relevantly affect the Hct predictions. Finally, the potential to predict the Hct of capillary DBS was demonstrated.

Conclusion: A commercially available NIR set-up was extensively and successfully validated, allowing non-contact Hct prediction of DBS with excellent accuracy and precision. This allows correcting for the Hct-based bias observed in partial-punch DBS analysis and the set-up of blood-plasma conversion factors, increasing the potential of patient-centric sampling.

### 3.10. Program Evaluations including Country Reports

P60. **Evaluation of the Expansion of NBS in the Netherlands**

Marelle J. Bouva, Rose E. Maase, Pim Vergeer, Wouter F. Visser, Samantha L. van der Beek, Ankie G.M. van Gorp and Eugenie Dekkers

Natl. Insitute for Public Health RIVM, Centre for Health Protection, 3720 BA Bilthoven, The Netherlands

Since 2018, the Dutch neonatal heel prick screening (NHS) program has expanded from 19 to 25 diseases.

In 2019, Carnitine palmitoyltransferase deficiency type I (CPT1), Methylmalonic acidaemia (MMA), and Propionic acidaemia (PA) were added. For screening on CPT1 and the combined screening for PA and MMA Flow Injection Analysis Tandem Mass Spectrometry (FIA-MS/MS) is used in combination with the NeoBase™ 2 Non-derivatized MSMS kit. For PA and MMA screening a second tier test was implemented. 

Screening for GALK-deficiency (added in 2020) uses total galactose (TGAL) concentration and galactose-1-phosphate uridyltransferase (GALT) enzyme activity. Implementation included the validation of an automated analysis of TGAL concentration.

For SCID screening (added in 2021) the first new laboratory application was implemented based on the detection of T-cell receptor excision circles (TRECs) by quantitative real-time polymerase chain reaction (qPCR). To limit the number of secondary findings an algorithm consisting of various covariant dependent cut-off values and repeat heel pricks was developed.

For screening of MPS-I (added in 2021), for the first tier the second new laboratory application was implemented: the marker, alpha-L-iduronidase (IDUA) activity, is measured with FIA-MS/MS. For all screen positive first-tier samples, a second tier test in-house multiplex UPLC-MS/MS method is used that measures the accumulation of the glycosaminoglycans (GAGs) heparan sulfate and dermatan sulfate. 

The first period of screening for the six most recent additions to the Dutch NHS programmhas been successful.

P61. **The Strength of a Neonatal Screening Program**

Natalia Cesari, Sofia Sanchez Campos, Carina Malmoria and Gisela Otero

IACA Laboratorios, Errores Congenitos Del Metabolismo, Bahia Blanca, Argentina

In newborns, congenital defects represent 5% of all births. Some of the alterations in genetic information cause the so-called Inborn Errors of Metabolism (IEM). These diseases are characterized by causing severe and irreversible damage, fundamentally at the neurological level, or by causing early death of the baby due to an acute metabolic crisis. In the neonatal period, the lack of specific symptoms is a challenge for the diagnosis; however, there are appropriate screening methods to detect these pathologies. The aim of these screening tests is to install an early effective treatment to prevent the outcoming consequences.

In this work, the protocol and guidelines of the Program for the Detection and Diagnosis of Congenital Diseases (ProDDEC), created in 2014 and reaching approximately 20,000 newborns per year from different parts of Argentina, are presented.

ProDDEC investigates seven diseases in the basic screening and around 54 diseases in the extended screening of newborn samples arriving from different provinces through its own specific logistics system. It includes all the confirmatory determinations including HPLC, GCMS, MSMS and molecular biology techniques. Each patient is studied individually, according to their characteristics at the time of birth. The presumed pathological results are treated urgently until the beginning of the patient’s treatment. Finally, biochemical and clinical follow-up is continued with the voluntary help of a multidisciplinary team.

Family reports and the observation of improvements in the quality of life of patients with IEM reflect the importance of neonatal screening programs and the need to expand the availability of laboratory determinations to detect a greater number of diseases and prevent their evolution.

P62. **Outcome of Dried Blood Spot Screening of Older, Previously, Unscreened, Children within the Newborn Screening Program in Sweden**

Lene Sörensen and Rolf Zetterström

Karolinska University Hospital, Centre for Inherited Metabolic Diseases, Stockholm, Sweden

Sweden has had a national newborn screening program using dried blood spots (DBS) since screening for PKU was introduced 1965. With the introduction of SCID screening in 2019, 25 diseases are now included in the program.

In Sweden, older, not previously screened, children are also offered screening through the national DBS screening program. Most commonly, these children are immigrants or international adoptions. All children younger than 18 years have been offered this test by our laboratory for a long time. Now there is a recommendation from the National Board of Health and Welfare that all children younger than nine years should be offered screening within this program. The PKU laboratory however still offers DBS screening to all children, i.e., any individual up to 18 years of age.

Between the first of January 2010 and the 31st of December 2020, 38,300 children who were not newborn, defined as older than 30 days of age at the time of sampling, were screened through the DBS screening program. During the same period, almost 1.3 million newborns were screened.

In this group of children, 59 were diagnosed with one of the 25 diseases. This corresponds to an overall incidence of 1:650. This is almost twice as high as the overall incidence in the newborn population, which for the same period was 1:1200.

The age at diagnosis for the older children ranged from six weeks to 15 years of age. The largest group were patients with PKU, followed by patients with hypothyroidism and CAH. Both very mild and more severe forms of the diseases were found.

In conclusion, even though the numbers are small, and it is a challenge for some disorders to tell what should be classified as a screen positive case, it is evident that it is a worth-while endeavor to offer DBS screening to older, previously unscreened, children in Sweden.

P63. **Current Status of Newborn Screening in Southeastern Europe**

Urh Groselj, Vanesa Koracin, Matej Mlinaric, Ivo Baric, Ian Brincat, Maja Djordjevic, Ana Drole Torkar, Ksenija Fumic, Mirjana Kocova, Tatjana Milenkovic, Florentina Moldovanu, Vjosa Mulliqi Kotori, Michaela Iuliana Nanu, Ziga Iztok Remec, Barbka Repic Lampret, Dimitrios Platis, Alexey Savov, Mira Samardzic, Biljana Suzic, Ildiko Szatmari, Alma Toromanovic, Mojca Zerjav Tansek and Tadej Battelino

University Children’s Hospital Ljubljana, University Medical Centre Ljubljana, Faculty of Medicine, University of Ljubljana, Department of Endocrinology, Diabetes and Metabolic Diseases, Ljubljana, Slovenia

Newborn screening (NBS) programs in the region of Southeastern Europe are in general non-harmonized with other developed European countries. In 2013/2014 the first survey was conducted to assess the main characteristics of NBS programs among 11 countries from the region (including Albania, Bulgaria, Bosnia and Herzegovina (BIH), Croatia, Kosovo, Macedonia, Moldova, Montenegro, Romania, Serbia and Slovenia) with a cumulative population of 52.5 million at that time. Plans for the future expansion and development of the programs were also given. At that time screening for phenylketonuria was not introduced in four out of 11 countries, while screening for congenital hypothyroidism was not present in three of them. None of the countries had an expanded NBS program for inborn errors of metabolism.

The survey was repeated in 2020. We invited the same 11 countries and added Cyprus, Greece, Hungary and Malta to the group. The aims of the survey were to assess the current state of the NBS programs, to evaluate the development and change in the period. We also wanted to identify the main obstacles in implementing an expanded NBS and/or reaching a wider population screening coverage. The responses were collected form 12 countries with a cumulative population of approximately 68.5 million.

The results of the survey showed modest improvement of the situation during this period. Congenital hypothyroidism was now introduced in 11 of the 12 countries. Phenylketonuria was not screened for in four of participating countries. An expanded NBS program using tandem mass spectrometry was successfully implemented in Croatia and Slovenia.

The current status of NBS programs in Southeastern Europe is very heterogeneous, underdeveloped compared to Western Europe or even non-existent in some of the countries. We suggest establishing an international task-force to assist with implementation and harmonization of basic NBS services where needed, further cooperation between the countries and creating common guidelines for NBS programs.

P64. **The Czech Republic: Report of Newborn Screening Results (2010–2020)**

Felix Votava, Petr Chrastina, Viktor Kožich, Karolina Pešková, Tomáš Adam, David Friedecký, Eva Hlídková, Hana Vinohradská, Monika Hedelová, Andrea Holubová, Milan Macek Jr., Veronika Skalická, Renata Gaillyová and Iveta Valášková

3rd Faculty of Medicine, Charles University and University Hospital Kralovske Vinohrady Prague, Pediatrics, Prague, Czech Republic

Nationwide newborn screening (NBS) in the Czech Republic covers the entire newborn population and since 2010 targets detecting congenital hypothyroidism (CH), congenital adrenal hyperplasia (CAH), cystic fibrosis (CF), phenylketonuria/hyperphenylalaninemia (PKU/HPA), leucinosis (MSUD), glutaric aciduria type I (GA I), isovaleric aciduria (IVA), medium chain acyl-CoA, long chain 3-hydroxyacyl-CoA and very long chain acyl-CoA dehydrogenase deficiency (MCADD, LCHADD and VLCADD), carnitine palmitoyltransferase I and II deficiency (CPTD I and II) and carnitine-acylcarnitine translocase deficiency (CACTD). Since VI/2016 the Czech NBS targets in addition argininemia (ARG), citrulinemia (CIT), biotinidase deficiency (BTD) and two homocystinurias (CBS and MTHFR deficiency). Here we report data for the period I/2010-XII/2020.

Methods: A total of 1,225,358 newborns (and a subset of 517,863 after expansion in VI/2016) were screened by use of fluoroimmunoassay for thyreotropin, 17-hydroxyprogesterone and immunoreactive trypsinogen (IRT). The CFTR gene (32 and later 50 mutations) was analyzed in 11,857 (0.97%) blood spots with the highest IRT levels. Amino acids and acylcarnitines were analyzed by tandem mass spectrometry, analyte ratios and second tier test for total homocysteine were also used. Biotinidase activity was determined by fluorimetric assay.

Results: We detected 1097 patients (cumulative detection rate 1:1117). The screening prevalence of particular disorders was as follows: CH 1:3003; CAH 1:12,013; CF 1:6284 (CF-SPID not included with a prevalence of 1:22,279); PKU/HPA 1:5214; MSUD 1:94,258; MCADD 1:22,692; LCHADD 1:87,526; VLCADD 1:245,072; GA I and IVA 1:204,226; CIT and CBS 1:517,863; BTD 1:9085 (including partial deficiency). 

Conclusion: NS is an effective approach for presymptomatic detection of serious rare diseases. Further optimization is needed. Pilot study of SMA and SCID is planned from 2022. 

(Supported by: PROGRES Q36 for FV and MH CZ-DRO-VFN64165).

P65. **The Status of Newborn Screening in Finland—Country Report**

Riikka Kurkijärvi

Turku University Hospital, Finnish Newborn Screening Centre, Turku, Finland

Newborn screening in Finland began at the turn of the 1970s and 1980s with screening for congenital hypothyreoidism from umbilical cord blood. This screening, decentralized to local laboratories, is still ongoing.

Screening for CAH and about 20 metabolic diseases from heel prick samples was introduced in 2015. In May 2018, it covered all districts of the country. This blood spot screening is centralized to one screening laboratory operating in Turku University Hospital and is based on oral informed consent. The coverages in 2019 and 2020 were 98.7% and 99.2%, respectively. Heel prick samples have been taken at the age of 48-120 h until March 2020 when sampling from the age of 36 h was allowed due to the ongoing pandemic. Screening results have been reported at the median age of 7 days.

At the end of 2020, a total of 250,000 Finnish babies had been screened. As in previous reports, PKU was found to be exceptionally rare in Finland, since only five cases of mild hyperphenylalaninemia and a PTPS deficiency were identified through the screening. Additionally, MCADD seems to be less common than elsewhere in Europe whereas diseases such as LCHADD, ASA, and tyrosinemia type 1 seem to be more common than in many other countries.

A SCID screening pilot was initiated in the Turku and Helsinki areas in 2019 and SCID screening will be extended nationwide in the near future. There has lately been discussion about SMA screening as well. Change of MSMS instrumentation and the consequent introduction of biochemical 2nd tier tests are currently in progress.

Data communication between maternity hospitals and the screening center has allowed the direct transfer of demographic and covariate data using barcodes in the screening cards without the need for manual entry. Similarly, screening results are transferred electrically to local hospitals and parents. Follow-up of the screening program has proven to be challenging. Data protection regulations and the lack of registries have made data collection more difficult.

P66. **UK Newborn Blood Spot Screening Update**

Stuart Moat

University Hospital of Wales, Medical Biochemistry, Immunology & Toxicology, Cardiff, UK

During the period 2019–2020 the UK (England, Scotland, Wales, and Northern Ireland) screened a total of 719,163 infants for nine conditions. The following number of infants were referred for further evaluation; PKU (106), MCADD (61), MSUD (4), IVA (26), GA1 (14), homocystinuria (7), congenital hypothyroidism (614), CF (284) and sickle cell disorders (262). PerkinElmer 226 collection devices are used in all 4 UK countries.

The UK National Screening Committee (UK NSC) recommended that screening for SCID should be evaluated and this is due to commence in September 2021. The evaluation will compare two different analytical technologies (ImmunoIVD SPOT-it™ & PerkinElmer EnLite™) for screening across six laboratories in England. Screening for SCID will be evaluated over a 2 year period (~790 K infants) with a further 18 months of screening after the evaluation to allow the UK NSC to consider the evidence and make a recommendation about whether screening for SCID will become part of the UK screening program.

In 2015, guidelines were introduced for the standardization of blood spot quality acceptance/rejection criteria to produce more accurate avoidable repeat rates that were comparable across the UK. To assess any variability in sample acceptance/rejection, two sets of color images containing both good and poor quality samples were produced and distributed to the 16 UK laboratories for assessment. The results demonstrated that there is inconsistency in applying the current acceptance criteria. A task and finish group has been formed with the aim to establish a minimum standard for blood spot quality, to describe a consistent way to introduce and maintain these standards without increasing the avoidable repeat rate to unacceptable levels.

P67. **Guiding the Worldwide Newborn Screening Community: An Update on CLSI Products for Newborn Screening Programs**

Amy Gaviglio

CDC/APHL, Genetic Counseling, Minneapolis, MN, USA

The Clinical and Laboratory Standards Institute (CLSI) provides standards and guidelines for professionals, developed through a unique consensus process that incorporates the values of inclusiveness, excellence, responsiveness, integrity, and teamwork. The collaborative approach includes balanced representation from members of industry, government, and health care professions throughout the world.

CLSI has facilitated the development of Newborn Screening (NBS) standards and guidelines since 1988 when the first NBS standard, Blood Collection on Filter Paper for Newborn Screening Programs, was published. Since 1988, CLSI’s NBS standards have grown to eight, with more currently in development. in the last two years alone, four NBS standards have been revised or developed. To date, five more are in the process of being revised and developed. These standards cover a wide variety of NBS topics and discuss pre-analytical, analytical, and post-analytical phases of the NBS system. 

This presentation will provide an overview of the topics covered by these standards, including any changes to guidance, and a discussion of available ancillary products. Specifically, this presentation will provide an overview of the following guideline topics:

- Blood Collection on Filter Paper
- NBS Follow-Up
- NBS for Preterm, Low Birth Weight, and Sick Newborns
- NBS for the following diseases:
  Cystic Fibrosis
  SCID and Other Related Immunodeficiencies
  Hemoglobinopathies
  X-linked Adrenoleukodystrophy
  Congenital Hypothyroidism
  Congenital Adrenal Hyperplasia
  Galactosemia
  Spinal Muscular Atrophy

Ongoing and future initiatives from CLSI will also be discussed, inclusive of terminology harmonization efforts, cost analysis in NBS, and additional ancillary products for CLSI readership. 

As the complexity of NBS continues to grow, it is more and more important for programs around the world to use CLSI standards and guidelines.

P68. **Enhancing Data-Driven Disease Detection in Newborns: A National Data Platform for Modernizing Newborn Screening Data Analytics and Interpretation**

Amy Gaviglio and Carla Cuthbert

CDC, Newborn Screening and Molecular Biology Branch, Atlanta, GA, USA

ED3N (pronounced “Eden”) is a National Newborn Screening Data Platform being developed by the Division of Laboratory Sciences Newborn Screening and Molecular Biology Branch (NSMBB).

The newborn screening system is facing an increase in data analytic challenges associated with ongoing expansion of the number of newborn screening diseases and the increased complexity of correlating biomarkers with disease risk and severity.

Through continuous collaboration between newborn screening programs and NSMBB, ED3N can assist programs by securely collecting, processing, and analyzing demographic, biochemical, molecular, and clinical data in near real-time. Ultimately, the goal of ED3N is to aid programs in assessing risk of disease at the time of screening. The presentation will discuss the overarching infrastructure and planned deployment of ED3N, which uses an interconnected modular approach as outlined below:

Biochemical module:

- Development and use of laboratory testing harmonization techniques to allow for aggregation of data across various platforms
- Use of machine learning for predictive algorithms to improve risk assessment
- Molecular module
- Development of streamlined bioinformatics pipeline with structured variant interpretation capabilities
- Newborn screening-specific workflows and genotype sharing across programs
- Linkage to biochemical and clinical module for analysis o genotype-phenotype relationships
- Clinical module
- Use of standard case definitions
- Collection of diagnostic data from both NBS programs and specialists

The newborn screening system generates an immense amount of data that is housed in programmatic data silos—without interoperability and aggregation, we cannot leverage the power of connected data for improving disease detection and understanding.

P69. **The BURDEN Study: Retrospective Metabolite Screening of Inborn Metabolic Diseases in Deceased Children**

Emmalie Jager, Willemijn van Rijt, Klaas Bijsterveld, Pim de Blaauw, Fjodor van der Sluijs, Ronald Maatman, Peter Schielen, Terry Derks and Rebecca Heiner-Fokkema

University of Groningen, University Medical Center Groningen, Beatrix Children’s Hospital, Section of Metabolic Diseases, Groningen, The Netherlands

Introduction: At least 43 Inborn Metabolic Diseases (IMD) associated with sudden infant death (SID) or Reye syndrome, of which many present at neonatal age, are detectable using metabolite analysis in dried blood spots (DBS). They are considered treatable. The BURDEN study (Bloodspot metabolite analysis in Unexpected Rapid DEaths before Neonatal screening) was initiated to investigate the prevalence of IMD in Dutch children who died in early childhood.

Methods: Stored neonatal DBS of deceased children under five years of age between 2013–2017 were requested from the Dutch National Institute for Public Health and the Environment (RIVM). In 1569 DBS, acylcarnitines and amino acids concentrations were analyzed using (LC-)MS/MS. Outliers were detected using a non-parametric method on log-transformed data, and principal component analyses. Two independent reviewers assessed whether the metabolite results of the outliers were suspect for an IMD.

Results: 270 (17%) abnormal profiles were identified; 166 (11%) were selected as possibly indicative of an IMD. Few profiles appeared (highly) specific for an underlying IMD, including tyrosinemia, glutaric aciduria, peroxisomal disease and citrullinemia.

Discussion/conclusion: Metabolite analyses in stored DBS of deceased children revealed a few specific IMD profiles. Interpretation was complicated, due to metabolite instabilities and lack of positive controls. Confirmatory genetic analysis is warranted, and will provide additional information on the prevalence of IMD and on optimal screening protocols after unexpected childhood death.

### 3.11. Miscellaneous

P70. **Global Impact of COVID-19 on Newborn Screening Programs**

Urh Groselj, Vanesa Koracin, J. Gerard Loeber, Matej Mlinaric, Tadej Battelino, James R. Bonham and COVID-NBS ISNS Global Network

University Children’s Hospital Ljubljana, University Medical Centre Ljubljana, Department of Endocrinology, Diabetes and Metabolic Diseases, Faculty of Medicine, University of Ljubljana, Ljubljana, Slovenia

The global pandemic of coronavirus disease 2019 (COVID-19) has presented extraordinary disruption to healthcare services and exposed them to numerous challenges. Newborn screening (NBS) programs were also affected, but only scarce data exist on the impact of COVID-19 on NBS.

We conducted an international survey to assess the global impact of COVID-19 on NBS with the main aim to gather experiences of the COVID-19 pandemic from a large and representative number of NBS centers worldwide.

Forty-three newborn screening centers from 38 countries took part in the survey. The results of our study showed that COVID-19 impacted the NBS programs, at least partially, in 29 out of 38 responding countries. The majority of the screening centers experienced a broad spectrum of difficulties and 65.7% were affected most prominently in the second wave of the pandemic. Delays and unreliability in the postal service as well as flight cancellations caused delays in samples arriving into screening centers and with the provision of laboratory equipment and reagents. The availability of laboratory staff was reduced due to infection, quarantine or reassignment within the healthcare facility. Sample collection at home, second-tier tests and follow-up were also affected. Social restrictions and the interruption of public transport added to these difficulties. Telemedicine emerged as important tool for maintaining outpatient care while limiting direct patient contact. The attention of the public health systems generally shifted from the provision of NBS services to COVID-19 related care. Only a limited number of centers managed to retain a fully functioning NBS program. Good practice examples were performed in Victoria, Australia and the UK.

The long-term effects of the pandemic on the well-being of children with disorders typically diagnosed by NBS remains to be reported. As the pandemic might be ongoing or could reoccur in future years, it might be useful to develop guidelines to protect these valuable services.

